# Supplementary material for: Allyship in Residency: An Introductory Module on Medical Allyship for Graduate Medical Trainees
Source: MedEdPORTAL. 2021 Dec 20;17:11200. doi: 10.15766/mep_2374-8265.11200 (PMC8685188; doi:10.15766/mep_2374-8265.11200)
Supplement: Supplementary file 1 — Facilitator Guide.docxAllyship in Residency Module.pptxCase Studies.docxEvaluation Form.docx [file mep_2374-8265.11200-s001.zip › D. Evaluation Form.docx]

**Allyship in Residency: An Introductory Module on Medical Allyship for Graduate Medical Trainees**

**Pre-Test**

**Part I. Demographic Information**

1. **What is your ethnicity? (Choose all that apply):**

- White or Caucasian
- Black, African-American, or African
- American Indian or Alaska Native
- Asian/Asian American
- Native Hawaiian or Pacific Islander
- Latinx or Hispanic
- Something Else _________________

1. **What is your gender identification?:**

- Male
- Female
- Transgender Male
- Transgender Female
- Gender Fluid
- Gender Queer or Gender Non-Conforming
- Prefer not to say
- Something Else _________________

1. **What is your sexual orientation?:**

- Straight
- Gay
- Lesbian
- Bisexual
- Pansexual
- Demisexual
- Queer
- Asexual
- Prefer not to say
- Something Else ________________

1. **What is your religious identification?:**

- Christian
- Catholic
- Jewish
- Muslim
- Mormon
- Buddhist
- Hindu
- Agnostic
- Atheist
- Prefer not to say
- Something Else ________________

1. **Are you a first-generation college graduate? (neither parents graduated from a four-year college or university:**

- Yes
- No

1. **Do you Identify as a person with disabilities?:**

- Yes
- No

1. **Did you receive a Pell Grant during your undergraduate education?:**

- Yes
- No

1. **We know you may be undecided, but to the best of your ability please answer the following question: At this point in your career, what is your career interest? (Choose all that apply):**

- Non-Academic Clinical Practice
- Academic Faculty: Clinical Teaching and/or Clinical Research
- Academic Faculty: Basic Science Teaching and/or Clinical Research
- Governmental Work
- Industry: Private Sector Work
- Something Else ________________

**Part II. Self-Assessment**

1. **Please rate how much confidence you have in your ability to be an ally to patients, medical students, and colleagues? 0 = no confidence, 4 = complete confidence**

- 0
- 1
- 2
- 3
- 4

1. **What is your level of knowledge regarding the concept of allyship?**

- Extremely knowledgeable
- Very knowledgeable
- Moderately knowledgeable
- Slightly knowledgeable
- Not knowledgeable at all

1. **What previous exposure, if any, do you have regarding allyship education? Select all that apply.**

- Trainings/Workshops Readings (Books, Articles, Memoirs, etc.)
- Media (Ted Talks, Education Videos, Interviews, etc.)
- Social Media (Facebook, Instagram, etc.)
- Other Sources
- I do not have any previous exposure to allyship education

1. **Rate how you feel about the following statement: "Allyship training and cultural competency training should be a required part of residency curriculum"**

- Strongly Agree
- Somewhat Agree
- Neither Agree nor Disagree
- Somewhat Disagree
- Strongly Disagree

1. **Which of the following ways are you most likely to respond to bias or discrimination within residency?**

- Confide in a colleague
- Confide in a supervisor/superior
- Address in the moment by speaking up
- Address later to person being biased
- Address later to person who experienced bias
- Ignore it
- Something Else

**Part III. Objective Assessment**

1. **What is the definition of Allyship?**

- Mutual trust and friendship among people who spend a lot of time together
- Speaking or acting in support of an individual or cause, particularly someone who intervenes on behalf of a person being attacked or bullied
- Supportive association with another person or group; more specifically, with members of marginalized or mistreated group to which one does not belong
- Helping another individual or group of individuals commit an action or effecting change

1. **What are ways to be an effective ally towards patients, medical students, and/or colleagues**

- Always strive for personal allyship above other forms of allyship
- Confront your own implicit biases
- Striving for equity rather than equality
- A and B
- B and C

1. **What does allyship look like across different communities?**

- Generally similar across different ethnicities, but always different across religious affiliations
- Always the same among members of a given community
- May be different across members from similar communities
- Partly different across different sexual orientations, but never different across individuals who share the same ethnicity

**Allyship in Residency: An Introductory Module on Medical Allyship for Graduate Medical Trainees**

**Post-Test**

**Part I. Self-Assessment**

1. **Please rate how much confidence you have in your ability to be an ally to patients, medical students, and colleagues? 0 = no confidence, 4 = complete confidence**

- 0
- 1
- 2
- 3
- 4

1. **What is your level of knowledge regarding the concept of allyship?**

- Extremely knowledgeable
- Very knowledgeable
- Moderately knowledgeable
- Slightly knowledgeable
- Not knowledgeable at all

1. **Rate how you feel about the following statement: "Allyship training and cultural competency training should be a required part of residency curriculum"**

- Strongly Agree
- Somewhat Agree
- Neither Agree nor Disagree
- Somewhat Disagree
- Strongly Disagree

1. **Which of the following ways are you most likely to respond to bias or discrimination within residency?**

- Confide in a colleague
- Confide in a supervisor/superior
- Address in the moment by speaking up
- Address later to person being biased
- Address later to person who experienced bias
- Ignore it
- Something Else

**Part II. Objective Assessment**

1. **What is the definition of Allyship?**

- Mutual trust and friendship among people who spend a lot of time together
- Speaking or acting in support of an individual or cause, particularly someone who intervenes on behalf of a person being attacked or bullied
- Supportive association with another person or group; more specifically, with members of marginalized or mistreated group to which one does not belong
- Helping another individual or group of individuals commit an action or effecting change

1. **What are ways to be an effective ally towards patients, medical students, and/or colleagues**

- Always strive for personal allyship above other forms of allyship
- Confront your own implicit biases
- Striving for equity rather than equality
- A and B
- B and C

1. **What does allyship look like across different communities?**

- Generally similar across different ethnicities, but always different across religious affiliations
- Always the same among members of a given community
- May be different across members from similar communities
- Partly different across different sexual orientations, but never different across individuals who share the same ethnicity

**Part III. Comments**

**Please answer the following questions:**

1. What did you like most about this workshop?
2. What suggestions do you have to improve this workshop?
