# Supplementary material for: Allyship in Residency: An Introductory Module on Medical Allyship for Graduate Medical Trainees
Source: MedEdPORTAL. 2021 Dec 20;17:11200. doi: 10.15766/mep_2374-8265.11200 (PMC8685188; doi:10.15766/mep_2374-8265.11200)
Supplement: Supplementary file 1 — Facilitator Guide.docxAllyship in Residency Module.pptxCase Studies.docxEvaluation Form.docx [file mep_2374-8265.11200-s001.zip › A. Facilitator Guide.docx]

**Allyship in Residency: An Introductory Module on Medical Allyship for Graduate Medical Trainees**

**Module - Facilitator Guide**

**Goal**

The goal of this module is to educate graduate medical trainees about the topic of allyship and provide a foundation for which trainees may use allyship within their clinical practice. Both definitions and tools will be provided throughout the module. Identities include, but are not limited to, LGBTQ+, Latinx, Indigenous, Black, Chronically Ill and Differently Abled, Undocumented, Female-Identifying, Asian-American, Non-English Speaking, and Muslim Communities. This module is intended for trainees of all identities.

NOTE: facilitators are encouraged to reach out to someone more knowledgeable if they feel unequipped to fully discuss a certain identity. Additionally, if facilitators are unable to answer questions, we encourage discussing with participants that you do not know the answer and offering to follow up or connect participants with someone who has more expansive knowledge.

No one is an expert on every topic, and therefore it is important to embrace the process of continuing to grow as an ally and reach out to those with more experience.

**Required Materials**

- Allyship in Residency Module
- Allyship in Residency Case Studies
- Allyship in Residency Facilitator Guide
- Allyship in Residency Evaluation Form
- Computer Projection or Virtual Meeting Link

**Workshop Objectives**

- Define Allyship
- Understand the ways to be an effective ally towards:
  - *Patients*
  - *Medical Students*
  - *Colleagues*
- Understand how allyship differs across communities.

**Suggested Agenda and Timetable**

- Pre-workshop evaluation: 2-3 minutes
- Slides 1-3: Introduction, Objectives, Agenda - 3 minutes
- Slides 4-7: Allyship Definition and Forms of Allyship - 4-5 minutes
- Slides 8: Health Equity Definition - 2 minutes
- Slides 9: Tools for Allyship - 2 minutes
- Slides 10-22: Allyship Across Different Communities - 20 minutes
- Slides 23: Intersectional Tips for Allyship – 2 minutes
- Slides 24: Relevancy to Residency - 3 minutes
- Slides 25: Minority Tax Definition – 2 minutes
- Slide 48: Why Does This Matter - 2 minutes
- Case Studies – 30 minutes
- Questions – 2-5 minutes
- Post-workshop evaluation: 2-3 minutes

**Slide Instructions**

Slide 1: Introduction and Title Slide

*Welcome to “Residency in Allyship”, a curriculum that will serve as an introduction to allyship as it relates to Residency.*

**Prior to presentation, please ensure you have inserted facilitator names, titles (if applicable), pictures, and pronouns to the slide, using the template provided.**

*Facilitators should introduce themselves, their title/institution, and pronouns to audience.*

**Start with a land acknowledgement, a Traditional Native American practice. Ensure prior research about what lands the session is inhabiting; please visit** [**https://native-land.ca/**](https://native-land.ca/)**. An example below:**

 “*We want to acknowledge that we are presenting, meeting, and gathering today on the traditional land of the [list tribal nations here, if applicable]* peoples of past and present”*

Slide 2: Workshop Commitments

Before beginning the presentation, read the commitments for this session, so that learners are aware and oriented to the safe space that you are trying to create.

*We ask that you make the following commitments to yourself and one another. Doing this will help you grow and learn the most from this session. These include: Strive for intellectual/cultural humility. Be open to challenging ideas.*

- *Pay attention to your reactions and use them as entry points for gaining deeper self-knowledge.*
- *Recognize how your identity informs your perspective.*
- *Whenever you think, “I already know something”, ask yourself, “how can I take this deeper?” Or “How am I putting into practice what I already know? Or at all?”*
- *Know that being an ally means you are growing and are not expected to know everything!*

Ask learners to remain open and honest during this workshop. Before proceeding, provide participants with the opportunity to ask questions or discuss concerns as they pertain to the workshop commitments.

Slide 3: Workshop Learning Objectives

Read the following learning objectives with the group of learners.

*At the end of this workshop, our goal is for you all to be able to do the following:*

- *Define Allyship*
- *Understand the ways to be an effective ally towards:*
  - *Patients*
  - *Medical Students*
  - *Colleagues*
- *Understand and learn methods of putting allyship into action*
- *Understand how allyship differs across communities.*

Again, provide participants with the opportunity to ask questions or discuss concerns as they pertain to the workshop learning objectives.

Slide 4: What is Allyship

*While some of you have probably heard of Allyship before, we would like to take a moment to give our set definition that we will be using throughout this workshop. While most individuals are perhaps most familiar with allyship within the context of the LGBTQ+ communities, allyship can, in fact, be used in a broader sense and more in line with the intersectionality of identities and communities that often occurs.*

***Allyship*** *is defined as a supportive association with another person or group; more specifically, with members of marginalized or mistreated group to which one does not belong.*

*Now that we have discussed what allyship is, let’s take some time to discuss what allyship is not.*

Slide 5: What Allyship is Not

*While Allyship can be many things, there are also common behaviors and definitions that some may consider to be allyship but are not. These include:*

- *Ally Theatre — allyship is not performative and should be affected by who sees your allyship (for example, limited to social media).*
- *A Free Pass — allyship is an active process – it is not finite. There is always room to grow, and sometimes you will continue to do things that are not quite allyship.*
- *“Helping” or “Saving” — Saviorism is dangerous – the goal is never to “save” someone or “help” those around us, as if they are inferior. It is, however, to engage and uplift in ways necessary to combat institution oppression.*
- *Being perfect — Mistakes are okay! The overall goal will continue to be to grow and improve. It’s a process, not a destination.*
- *A state of being —* ***allyship is a verb not a noun, meaning that it is an active, ongoing process that one must work to demonstrate and exemplify, rather than claim to be.***

Slide 6: Forms of Allyship

*Allyship is a process. There are many ways it evolves throughout a person’s life. Let’s talk about the stages and forms of allyship*:

- *Aspiring ally for self-interest*
  - *This form of allyship places the focus on the individuals in question, meaning that one is motivated to demonstrate often limited allyship because it involves someone they know or care about.*
  - *This involves allyship towards a specific person. For example, after bonding with a patient who experiences deafness and was recently admitted to the hospital for an extended period, a doctor goes home and looks up basic greetings in American Sign Language (ASL). Within the context of this example, this is done because the doctor cares about the patient specifically and wants to greet them in the morning so that they feel welcome and included while in the hospital.*
  - *However, this form of allyship often involves working over members of the target community. While it often serves as a starting point for many people, aspiring allyship for self-interest should evolve past the motivation to be an ally to a specific person.*
- *Aspiring ally for altruism*
  - *This form of allyship tends to focus on others such that the motivation for demonstrating allyship stems from the concern for the well-being of others. This often involves demonstrating allyship towards a target community because one intends to “help” a common group of people.*
  - *Using our same example from before, the doctor volunteers their time and donates money to the National Association of the Deaf, specifically because they feel obligated or motivated to “help” persons experiencing deafness. This involves working for members of the target community.*
  - *While this motivation to demonstrate allyship shifts away from the personal and individual and more towards broader communities, it often reinforces the notion of “us and them” and too easily blurs the line of allyship with saviorism.*
- *Aspiring ally for social justice*
  - *This form of allyship is the ultimate goal as it focuses on rebuilding the system, such that the motivation to demonstrate allyship stems from the knowledge that one’s own liberation is tied to the liberation of everyone and requires that we all must work together for the common benefit of* ***our*** *greater future.*
  - *Circling back to our example, a doctor takes the time to educate themselves on the challenges often faced by the community of persons who experience deafness works with community members to advocate for more inclusive and equitable practices at their institution in an effort to increase the accessibility of services available to persons who experience deafness.*
  - *The motivation to demonstrate allyship stems from dedication to the cause of social justice and involves working with members of the target community.*

*There is no shame or judgement wherever you are in your Allyship process. We encourage you to take a moment to reflect on where you are – honestly – and how you can continue to work through your Allyship journey.*

Slide 7: Aspiring Allyship for Social Justice

*As we just discussed, the ultimate goal of Allyship is to achieve Allyship for Social Justice. Let’s discuss some ways that we can address our own current behaviors, or common Allyship behaviors, and shift our focus to a more social-justice focused approach:*

- *Shift from striving for equality to striving for equity. Equality is treating everyone the same, while Equity on the other hand is giving everyone what they need to be successful. More on this in a moment.*
- *Let’s discuss the need to shift from identifying “at risk” individuals to acknowledging that the system is broken by design. we must scrutinize the systems of stratified privilege that make it such that certain individuals are labelled “at-risk” because of who they inherently are or the identities they possess.*
- *Shift from “color blindness” to self-examination. “Color-blindness” inherently ignores the existence of race-based difference, which in turn ignores the existence of systemic racism. These topics can often make people uncomfortable, which is why rather than ignoring them under the guise of “color blindness”, we need to critically examine why these topics may make us feel uncomfortable.*
- *Shift from “celebrating diversity” to advocating for systemic change and dismantling systems of entitlement and privilege.*
- *Shift from focusing on intent to focusing on impact. So often it is easy to focus on why we said or did something. Oftentimes our intentions may be non-malicious or positive in nature. However, rather than focusing on our intent, it is important to hold ourselves accountable for the impact that our words or actions had.*

**Please insert personal examples for each point that you have brainstormed prior to session. This will create connection with the audience, offer humility, and expand on what each definition means in the most relevant setting.**

Slide 8: Equity

*When discussion Allyship, it is important to also define and discuss equity. Many people often use “equality” and “equity” interchangeably – let’s parse this out and outline differences.*

*Equality is treating everyone the same. Equity is providing everyone what they need to be successful. This means assessing people where they are and seeing what they need to be equally successful to the person next to them.*

*Intending to enhance equity is not enough. Outcomes are really important when discussing equity because intentions do not guarantee results.*

*Assess community needs and partner to enhance equity systematically and strategically in your sphere of influence. Remember to continually work to improve the efficiency and effectiveness of programs and policies in enhancing equity*

Slide 9: Tools for Allyship

*Now that we have defined Allyship and Equity, let’s talk about a practical tool that can be applied to situations regarding Allyship moving forward*.

*A practical tool that we can use when working toward allyship is “REAL TALK”*

*To practice* ***REAL TALK****, you will:*

- - *Realize Reality — Understand that reality looks different to different people with different life experiences and perspectives.*
  - *Embrace Listening to New Perspectives — take the time to hear what other people have to say and share about their experiences.*
  - *Assess Yourself: What Are You Doing? — Scrutinize your daily practices and ask yourself how you can change for the better to deconstruct oppressive practices.*
  - *Lean Towards Vulnerability — Allow yourself to be open and unguarded in these discussions. By bringing vulnerability to the conversation, you show that you willing to learn and grow.*
  - *Take Into Consideration Historical Representations — Educate yourself on historical representations, including historical traumas and historical oppression that may have affected certain communities. Try to consider how this may affect how someone view’s the world or experiences life.*
  - *Attempt to Learn — Taking the time to educate yourself and being an open learner is an important central tenant of allyship. Educating yourself shows not only that you care, but it also enables you to better understand how you can more effectively demonstrate allyship towards certain communities.*
  - *Live as an Agent for Change — Embody allyship in all that you do. Do not limit your demonstration of allyship to certain spaces or in front of certain people.*
  - *Keep At It — Again, allyship is a verb. Continue to grow and learn every day. There is always more work to be done.*

Slide 10: Allyship Across Communities

*We have now discussed definitions and tools as they relate to the general idea of Allyship.*

*Let’s discuss specific ways to be an ally to different underrepresented identities in the medical community, with a focus on how we can immediately implement skills for community****at [insert institution/location/community]***

*Disclaimer: While we may belong to some of these communities, we certainly do not claim to speak for every and all underrepresented communities.*

*The best way to learn allyship skills is to use resources provided by each individual community.*

*It’s always helpful to dedicate time to exploring resources on your own – there are endless avenues to do this, including your local organizations, the internet, and social media.*

Slide 11: Allyship in LGBTQIA+ Communities

*The LGBTQIA+ Community represents individuals who are underrepresented in their sexual orientation and gender identification. Important things to remember in Allyship towards these communities includes:*

*Unlearn historical messaging — It’s important that we identify, unpack and challenge stereotypes and unconscious bias towards the community, and that starts with knowing a little history. Homosexuality was criminalized nationwide until 1962. It wasn’t until the mid-1980s (35 years ago) that gender affirming care and recognition of trans people began to be remotely accepted. Same sex marriage across the country wasn’t legalized until 2015.*

*Educate yourself — Read books, listen to podcasts, watch shows/movies by, or about, LGBTQ+ people.*

*Learn and use correct and inclusive language, including the following important terms to know and appropriately:*

- - *Gender Identity- how you, in your head, think about your identity – this is non-binary!*
  - *Sexual Orientation – who you are physically, emotionally, and mentally attracted to*
  - *Attraction – who you are attracted to*
  - *Biological Sex – this is biological, and often defined as “male” or “female”, although many sources believe this is non-binary (not just two options) – intersex is also defined under biological sex and is varying in presentation*
  - *Gender Expression – how you demonstrate your gender to those around you*

*Embrace those within the LGTBQIA+ communities — Recognize the intersectionality of the LGBTQ+ communities and embrace people of all colors, shapes, genders, and intersecting identities.*

*Speak up and intervene — Don’t tolerate anti-LGBTQIA+ rhetoric or statements expressed in your presence.​ Speak up on behalf of LGBTQIA+ people to help remove some of the burden from them directly.*

*Keep up with current events that relate to or impact the LGBTQIA+ communities and take action where appropriate — the LGBTQIA+ communities continue to face institutionalized oppression that requires ongoing activism.*

Slide 12: Allyship in Latinx Communities

*The Latinx Community represents individuals who are underrepresented in their ethnicity. Important things to remember in Allyship towards these communities includes:*

- *Seek, learn and affirm the histories of distinct Latinx communities — Latinx countries each have rich and unique experiences and histories that shouldn’t be disregarded and lumped into one under the term “Latinos’​.*
- *When appropriate, inquire about a person's background/heritage instead of making assumptions or generalizing. It is better to ask for clarification when appropriate rather than make assumptions about someone based on their presumed identity.*
- *Learn and understand the difference between the terms Hispanic and Latinx and diversity within these communities – Hispanic includes Spain but does not include Brazil. Latinx includes Brazil but does not include Spain. There are different contexts to use these terms.*
- *Why the x? – this de-genders the word. By doing so, you do not assume someone’s gender and remain gender-neutral. This exemplifies great intersectionality between the Latinx and LGBTQ+ community.*
- *Finally, do not make assume someone's first language or food preferences based on their heritage.*

Slide 13: Indigenous/Native Communities

*The Indigenous/Native Community represents individuals who are underrepresented in their ethnicity. Important things to remember in Allyship towards these communities includes:*

- *Learn a more nuanced view of US history that dispels myths and misconceptions and reclaims native truth (e.g., including resistance to injustices and colonization) —* *Traditional US history that we all largely ignores indigenous histories and erases the history of genocide, treaty violations, and atrocities committed against indigenous communities by the US government. ​It is important to unlearn this narrative surrounding indigenous communities in this country and recenter the narrative around indigenous voices.*
- *Next, Identify and actively acknowledge the land you live on —*[*https://native-land.ca/*](https://native-land.ca/) *is a great resource to identify the indigenous land that you are on today*
- *Be conscious of word choice — Avoid jokes about tribal names, dwelling places, historical traumas, socio-economic conditions,  or the use of stereotypes or Native “themed” mascots.*
  - *“Pow Wow”, “Spirit Animal”, “Indian giver”, etc. are rooted in appropriation from Indigenous culture*
- *Avoid and deconstruct continued appropriation — understandably, many tribal nations are wary of the appropriation of indigenous identity. Identify and call out appropriation of native identity and understand that many commonly appropriated elements of native culture hold deep spiritual.*

Slide 14-16: Allyship in Black Community

Slide 14

*The Black Community represents individuals who are underrepresented in their ethno-racial identity. Important things to remember in Allyship towards these communities includes:*

- *Seek, learn and affirm the histories of distinct Black communities in the US — the history of Black communities in this country is multifaceted and cannot be considered without also considering the lasting legacy of slavery, atrocities, and the hundreds of years of systemic racism that still persists today. However, while it is important to consider historical injustices as a framework for which we learn and affirm the histories of Black communities in the US, it is just as important to also highlight and uplift historical narratives of Black joy, Black success, and Black excellence — not in an effort to erase or ignore the intergenerational and pervasive oppression of Black communities, but rather, as an act of resistance against the forces of systemic racism and white supremacy that act to dismantle and oppress Black joy, Black success, and Black excellence.*
- *Considering this, it is therefore also important to understand the legacies of resistance to injustices that have uniquely affected Black communities, particularly those communities with deep historical legacies in the US.*
- *Next, understand that not everyone identifies as “African-American”, as identities can include: Black, African, African-American, Caribbean, Afro-Latinx, Afto-Asian, and more.*
- *Given this, learn how each person would like to be addressed If unsure, and* ***if relevant****: ask “What is your preferred identify term ethnically/racially*

Slide 15

*Given the long-standing oppressive history of the Black Community in the United States, we feel that it is important to provide additional tools when practicing Allyship towards this community:*

- *First, begin by confronting your racist tendencies and implicit biases*
- *Next, it is important to understand and acknowledge that systemic racism exists at every level of society*
- *Take the time to listen to Black voices and communities, learn from them, and educate yourself*
- *Speak and act out against police brutality, which includes advocating for systemic change, deconstructing and actively resisting the pervasive devaluation of black life, and dismantling systems of oppression that exist within the broader criminal justice system (for example, the school to prison pipeline).*
- *Finally, use your privilege (and your physical and monetary resources) to support Black people, issues, businesses and projects.*

*Most systems are built on oppressive policies and infrastructure that are rooted in the oppression, specifically, of the Black community. It’s important to always remember this in all situations.*

Slide 16

*Additionally, consider incorporating the term “BIPoC”. It stands for: Black, Indigenous, and People of Color. This term that recognizes, accepts, names and celebrates group experiences for people of color within the US context. It moves away from using "non-white", "minorities", and "POC". This places oppression and increased inequity of the Black and Indigenous at forefront of dialogue, an important first step in deconstructing systems and striving for social justice Allyship.*

Slide 17: Allyship in Chronically Ill and Differently Abled Communities

*The Chronically Ill and Differently Abled Communities represent individuals who are underrepresented for a variety of reasons and are often left out of the conversation surrounding diversity. Important things to remember in Allyship towards these communities includes:*

- *Remember that disability is a part of diversity — Do not overlook the unique experiences and perspectives of those within this community. Remembering to include these voices in conversations surrounding diversity, anti-oppression, representation, and equity is an essential part of intersectional allyship. Additionally, the communities of persons with disabilities are extremely diverse and comprised of many different people with intersecting identities.*
- *Use words with dignity – if it is necessary to discuss a person’s disability status, always refer to someone as “a person with [insert disability]” instead of phrases like “disabled” or “differently abled” – it’s important to put the person at the center, not the disability. Remember that words with dignity are constantly changing and evolving, and when appropriate, allow the persons and/or communities to model how they are to be referred to when necessary. The words with dignity that someone uses to describe themself may also vary from person to person.*
- *Next, work to view aids that enhance quality of life as more than just devices and remember that an individual is not defined by their use of an aid or lack thereof. With this in mind, address the person in question directly, not their companion or assistive aid.*
- *Ability/Disability does not define people but recognize that it may or may not be an important part of their identity.*
- *Learn about and implement universal design — remember that increasing accessibility starts with working to change attitudes within your organization or environment. Initiatives to increase accessibility should be motivated by a desire for equity, rather than by obligation or compliance standards.*
- *Finally, remember that disability does not mean inability — this means working with people living with disabilities to properly accommodate their needs, if any adjustment is even necessary, rather than assuming someone's disability defines their overall ability​.*

Slide 18: Allyship in Undocumented Communities

*The Undocumented Community represents individuals who are underrepresented in their citizenship, although there is often an ethno-racial component associated. Important things to remember in Allyship towards these communities includes:*

- *Learn and use the correct terms —rhetoric surrounding undocumented immigrants commonly dehumanizes and villainizes undocumented people. It is important to re-center the terminology used to define immigration around the humanity of the act.*
  - *Correct: Undocumented, patient, student, immigrant*
  - *No human is Illegal or Alien*
- *Learn the history of US immigrant policy and how it informs current rhetoric and policy and be vocal in your support.*
  - *Data show that immigrants are actually less likely to commit crimes than U.S. born citizens.*
  - *Undocumented in the U.S. CAN and often DO pay taxes and DO NOT receive any federal benefits such as Social Security, Supplemental Nutrition Assistance Program (SNAP), etc.*
- *Embrace the intersectionality of this group — undocumented people come from all over the world for a variety of different reasons.*
  - *Not all undocumented people are Latinx/Chicanx*
  - *Those who are undocumented in the U.S. migrate from all over the world including: South Korea, India, Canada, and more.*
- *Last and perhaps most important, recognize that undocumented communities are vital to the US and make substantial contributions to the country.*

Slide 19: Allyship in the Female-Identifying Community

*The Female-Identifying Community represents individuals who are oppressed due to their gender identity. Important things to remember in Allyship towards these communities includes:*

- *Listen to a diversity of womxn’s voices, which means embracing and promoting intersectional feminism. This requires recognizing that the female experience differs from person to person and other intersecting identifies may play a role in shaping said experiences. This means requires us to recognize that white feminism does not equal Feminism — women of color are often left out of the conversation regarding womxn/female rights. Given this, remember that Allyship towards female-identified communities also includes, among others:*
  - *BiPOC Womxn*
  - *Trans Womxn*
  - *Queer Womxn*
  - *Differently-Abled Womxn*
  - *Womxn observers of various of levels of religiosity/spirituality*
- *Highlight womxn’s voices at every opportunity, which can include confronting sexism, advocating for equal pay rates (the pay gap is still very prominent and varies based on ethno-racial identity), and recognizing the motherhood and family/work burden. While not all womxn want to be or have the ability to be a mother, parenthood is often not regarded as central to male identity but is subconsciously centered in the identity of females.*

Slide 20: Allyship in Asian/Asian-American Communities

*The Asian/Asian-American Communities represents individuals who are oppressed in their ethnicity. Important things to remember in Allyship towards these communities includes:*

- *Seek learn and affirm the histories of distinct Asian and Asian American communities and legacies of resistance to injustices that have uniquely affected them — recognize that the Asian and Asian American communities in the US are extremely diverse and represent many different ethnic and linguistic groups with distinct identities, cultures, and histories. Each of these groups have experienced historical and ongoing injustices and each have their own stories of resistance that deserve to be heard. Take the time to listen to these perspectives as one way to learn and educate yourself.*
- *Understand that the "Model Minority" myth, which refers to Asian and Asian American individuals as the “model” or “consummate” minority group, is a hurtful generalization and leads to erasure. This myth perpetuates the fallacy of the Asian and Asian American communities as a monolith, it reinforces the damaging notion that assimilation into the dominant white culture is the definition of American success, and it erases the perpetuation of injustice and racism against Asian and Asian American Communities.*
- *Especially following the COVID-19 Pandemic, unacceptable racist and violent acts against Asian and Asian American Communities have been sharply increasing. You can demonstrate your allyship by making sure to use the language supported by scientists and public health officials when referring to COVID-19, in addition to actively speaking up and denouncing discrimination and hate crimes by discouraging jokes and narratives that blame certain groups or countries for the spread of the virus.*

Slide 21: Allyship in Muslim Communities

*Muslim Communities represents individuals who are oppressed in the US based on their religion. Important things to remember in Allyship towards these communities includes:*

- *Start with rejecting the assumption that you know or understand everything about someone’s religion. Islam, like all world religions, is a highly diverse and multifaceted religion that is practiced differently by all different people all over the world.*
- *Importantly, take time to learn about issues and concerns of Muslim communities.*
- *When appropriate, you can tart conversations with colleagues and patients about their spiritual needs. For example, this can include ensuring that Muslim patients and colleagues have respected time and space for prayer and that considerations are made for Ramadan and other religious observations.*
- *Proactively foster environments that are safe, accepting, and supportive for women wearing hijabs or coverings. Within medicine, this can mean making sure that inclusive considerations are made in places such as the OR where scrubs and caps are worn.*
- *Finally, Get involved with movements outside of your religious heritage or join an interfaith discussion group to broaden your world view past your own spiritual or religious beliefs.*

Slide 22: Allyship in Non-English-Speaking Communities

*Non-English-Speaking Communities represent individuals who are underrepresented in their language. Important things to remember in Allyship towards these communities includes:*

- *There is a difference between interpreters and translators! The two terms should not be used interchangeably as they confer different resources. An Interpreter is a person specifically trained to interpret oral language from one person to another, while a translator is a person specifically trained to interpret written language from one person to another.*
- *Additionally, try to seek out services that assist in communication, such as iPads with interpreter services from every language available. Remember to seek services even if there is only a slight communication barrier*
- *Decentralize English as the expected norm and affirm that it is okay if someone does not speak English — let them know that they are safe within your care.*

Slide 23: Intersectional Tips for Allyship Across Communities

*We have discussed a lot of introductory information regarding specific demonstrations of allyship towards different communities. In order to help synthesize this together, we want to take this opportunity to mention the following points for allyship across communities. First, it is important to strive to promote anti-oppressive allyship in everything you do, in and outside of activist space. In order to do so, avoid generalizing feelings, thoughts, behaviors etc. to a whole group. Remember to continue to set anti-oppression goals and continually evaluate whether you are meeting them. The goal is to continue to grow. Finally, don't feel guilty — Feel motivated. Realizing that you are part of the problem doesn't mean you can't be an active part of the solution!*

Slide 24: How is this Relevant to Residency?

*Residency is the intersection of becoming the physician you want to be and the physician you are becoming. You are constantly provided the opportunity to directly work with many different groups, and autonomy to make decisions. This may include patients, medical students, and Attendings. We understand that these skills and objectives seem most applicable to patients, but there is plenty of opportunity to implement Allyship within your team at work. While the power dynamic may increase hesitancy in promoting Allyship directly towards Attendings, there is room to navigate that discussion in a way that is not accusatory or aggressive.*

*In regard to medical students, this is the perfect opportunity to model Allyship for the future generation of physicians. Additionally, you have the ability to directly intervene when you witness discrimination towards medical students on your service.*

*We hope you use Allyship to shape your experiences to advocate for the communities you work with and advocate for your own identities, while effecting systemic change.*

Slide 25: Minority Tax

*As you are working within your institution, it’s important to remember that your colleagues from underrepresented and oppressed identities often face more burden than what is seen at face value.*

*Specifically, the Minority Tax is known to be increased stress on such identities. It is defined as: "the tax of extra responsibilities on minority faculty in the names of effort to achieve diversity"*

- *This can look like:*
  - *Racial Disparity*
  - *Isolation Disparity – potentially feeling alone if no other colleagues share that identity*
  - *Mentorship Disparity – potentially struggling to find a mentor who reflects identity*
  - *Clinical Disparity – sacrificing clinical time to work on other initiatives, such as diversity initiatives and community service*
  - *Promotion Disparity – being missed for promotion due to lower research/clinical research because time was spent working towards advancing health equity (a very important need, which is often not as highly regarded in terms of promotion).*

*The responsibility to advocate for diversity and equity needs to shift from those facing inequity, to those who have power within the system – the allies.*

Slide 26: Why Does This Matter?

*Allies within medicine and healthcare needed now more than ever to address institutionalized oppression and injustices that continue to persist within the field. Oftentimes, structures have been put into place within medicine that perpetuate oppression and inequity. An example of this includes so-called “race-based corrections” within medicine, such as those for eGFR, VBAC, spirometry. Remember, race, and other forms of identity-based medical practices, are not founded on any biological evidence. Correlation does not equal causation, confounding factors often alter outcomes, and many times race is often used as a proxy for other factors, the most common one being the effects of systemic racism.*

*To learn more about how inequity exists within medicine, per specialty/system, visit this website to learn more:* ***raceandmedicine.com***

Slide 27: References

*Here are the references we used for this discussion – but we encourage you to do your own research!*

*Are there any questions before we move on to the Case Studies?*

**Case Studies – Facilitator Guide**

**Prior to Case Studies:**

1. **Introduce yourself, your pronouns, and how you self-identify**
2. **State a positive trait/characteristic about yourself that you will bring to the table as an ally**
3. **What you look forward to in your journey with allyship**

**Scenario #1**

**Allyship in Residency**

*You are the resident on service working with Dr. Smith. This morning, you have two new medial students working with you on your service that you are just meeting for the first time.*

Scenario:

During morning pre-rounds, you are greeted by two medical students new to your service. The first medical student introduces themselves and you cordially shake hands. The second medical student states: “Hi, my name is Sam, and my pronouns are she/her/hers”. You greet Sam cordially and make note of her pronouns.

After a morning of pre-rounding patients, you are joined by your attending, Dr. Smith. Both medical students introduce themselves, and Sam again states her pronouns. However, throughout the course of morning rounds, you notice that the attending keeps referring to Sam as “him” and “his”. Sam politely corrects Dr. Smith the first time, and is met with the response “My apologies, but these days everyone wants to be called something different. It’s hard to keep up”. He continues to misgender Sam for the rest of the day.

**Discuss your role as the Resident in this situation, and how you would choose to approach this scenario.**

**-**Would you choose to address this situation directly on rounds? If so, how?

There is no correct answer for this question – but more so multiple considerations. This can be tricky because the attending is your superior and therefore there is a power dynamic. Additionally, Sam may not want you to draw attention to the situation in that setting. However, it is important to find ways to reinforce Sam’s pronouns while on the floor.

-If you chose to not address on rounds, how would you address this situation after rounds?

Consider reaching out to Sam directly – and starting a private discussion about what happened, if she is okay, and what you can do to support her. It’s important for Sam to know that misgendering her is not okay, and that she has support.

-What are ways to approach this with Sam, the medical student?

Using REALTALK, this is a great opportunity to Assess Yourself and Lean Towards Vulnerability. By doing this, you can have an open and honest conversation with Sam about the impact that interaction had and ask ways that you can better support her moving forward. It’s important to elicit what is important to her in terms of actions moving forward – would she like you to talk to Dr. Smith in a non-confrontational way? What is her preferred way for you to handle this situation?

-What are ways to approach this with Dr. Smith, the attending?

This is a great opportunity to dismantle systems of entitlement, power, and privilege in a diplomatic way. When speaking with Dr. Smith in a private environment, work to educate him about the importance of using correct pronouns when speaking to others, including patients, medical students, residents, and colleagues. While this may seem like an uncomfortable situation – consistently misgendering and ignoring pronouns can alienate both medical students and future patients from feeling safe and supported in their environment. Try to relay to Dr. Smith the impact it may have on Sam, while advocating for change based on your discussion with Sam.

-How does allyship play a role in this scenario?

There is no right answer to this question!

**Scenario #2**

**Allyship in Residency**

*You are the resident working in a clinic. You are meeting a new patient, Tom, for the first time. Tom is a 10-year-old male. He is accompanied by his mother, Mary.*

Scenario:

This morning in clinic, you notice that a new patient, Tom, is on your schedule. When he arrives, you enter the room and introduce yourself to both Tom and his mother, Mary. Tom anxiously greets you and introduces both himself and his mother. Mary nods silently in your direction.

You ask what has been going on, and why they have come to clinic today, and Tom readily answers all your questions. When you address Mary about what has been going on, she again nods and smiles, and looks over to Tom who interjects and answers again. You begin to suspect that Mary may not be comfortable speaking English.

**Discuss your role as the Resident in this situation, and how you would choose to approach this scenario.**

-How would you approach a discussion with Tom and Mary to assess and discuss Mary’s ability to speak English? There is no correct way to approach this – it should be what feels more comfortable to you. A non-confrontational way to elicit this information would be simply say “Hey Tom, if it’s okay – I need your mom to answer some of these questions if that’s okay.” Based on Tom’s response, you can ask further why you may not or can’t speak directly with his mother, and from there gently bridge the conversation to language barrier.

-What would be appropriate next steps for providing care, if Mary is unable to speak English well?

It would be necessary to bring in interpreting services.

-What would be more appropriate – translator phone or interpreter iPad services? Why?

When able, iPad interpreter services are ideal. The create a more personalized conversation, allow for better interpretation and communication, and include nonverbal cues which are very important for discussing difficult topics.

-What would you do if Tom insists that it is okay for him to remain translating, as that is something he always does?

It is important to gently let Tom know that you are required to bring in interpreter services. However, it’s also to acknowledge that Tom does a great job and validate that he has done nothing wrong. It’s also important to let Tom know that they are safe with you as their provider, and you just want to make sure he gets the best care possible

-How does allyship play a role in this scenario? There is no right answer to this question!

-What are additional considerations that may be of concern for this patient?

It’s really important to consider fear and safety concerns for non-English speaking individuals. Especially in the times of enhanced immigration, customs enforcement, it is reasonable that families may feel hesitant to trust the systems in place to protect them. Additionally, there may be health literacy concerns that compound the lack of English. It’s important to be mindful of extra factors and work with compassion to meet Tom and Mary where they are at.

**Scenario #3**

**Allyship in Residency**

*You are the resident working on service, and you have called a consult from your colleague, Dr. Brooks, for your patient, John, with additional concerns. Since you have been with your patient for the past week, you decide to be present for the consultation.*

Scenario

John has been your patient for the last week, and you have noticed that he may have additional health needs that require consultation. John has been a compliant patient and is totally invested in addressing his health needs. You have developed a good rapport with John. You decide to call a consult, and your colleague Dr. Brooks is on service and is able to provide a consult in 15 minutes. You greet Dr. Brooks and enter the room with him.

Upon introduction, John seems visibly tense, and his demeanor is notably different than it has been for the last week. John asks if he may have a word alone with you, without Dr. Brooks.

When Dr. Brooks leaves the room, John states “I do not feel comfortable with an African American doctor. I want to be seen by another doctor” You are taken aback, because you have never heard John speak like this before.

**Discuss your role as the Resident in this situation, and how you would choose to approach this scenario.**

-How would you respond to John, after he has told you this information?

There is no right answer to this question. It is important to uphold patient autonomy – however, it is also important to reinforce that Dr. Brooks is a capable and equally skilled physician to any of his colleagues. If there are no other colleagues working besides Dr. Brooks, you must provide John with the option to delay potentially lifesaving care if he is uncomfortable with Dr. Brooks. However, again, it is important to strive for equity and not reinforce systemic biases that this patient may have.

-How would you relay this information to Dr. Brooks?

It is important to be honest with Dr. Brooks – and also ask how you can best support him in this situation. Using REALTALK, you should Realize Reality, and Attempt to Learn from Dr. Brooks what this experience is like for him, and what he would like you, as his colleague, to do moving forward to best support him in situations similar to these. After you’ve listened to Dr. Brooks perspective, you should work to Live as an agent for change, based on his needs.

-How does allyship play a role in this scenario?

There is no right answer!
