# Supplementary material for: Allyship in Residency: An Introductory Module on Medical Allyship for Graduate Medical Trainees
Source: MedEdPORTAL. 2021 Dec 20;17:11200. doi: 10.15766/mep_2374-8265.11200 (PMC8685188; doi:10.15766/mep_2374-8265.11200)
Supplement: Supplementary file 1 — Facilitator Guide.docxAllyship in Residency Module.pptxCase Studies.docxEvaluation Form.docx [file mep_2374-8265.11200-s001.zip › B. Allyship in Residency Module.pptx]

## Slide 1
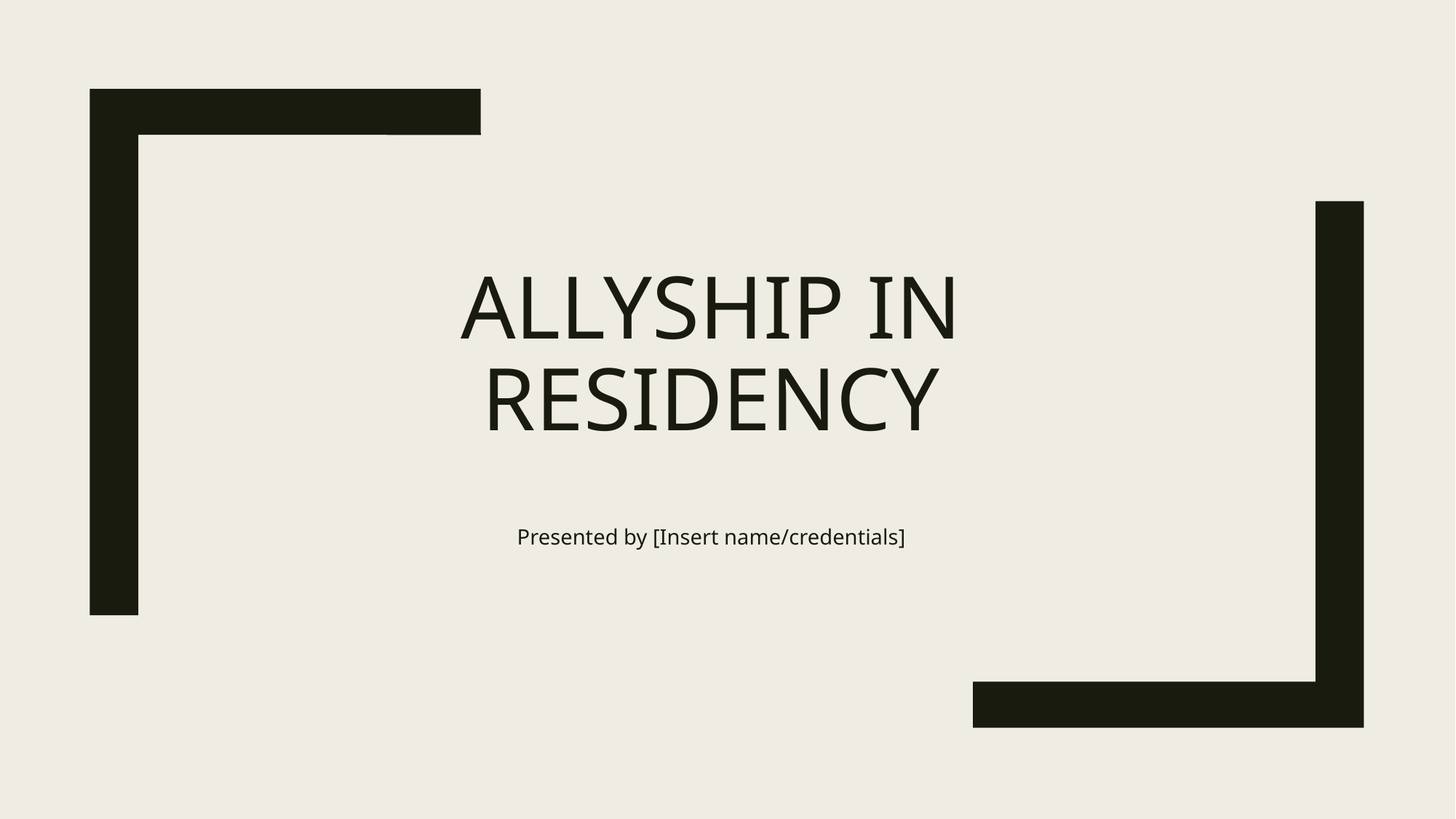

# Allyship in Residency
Presented by [Insert name/credentials]

## Slide 2
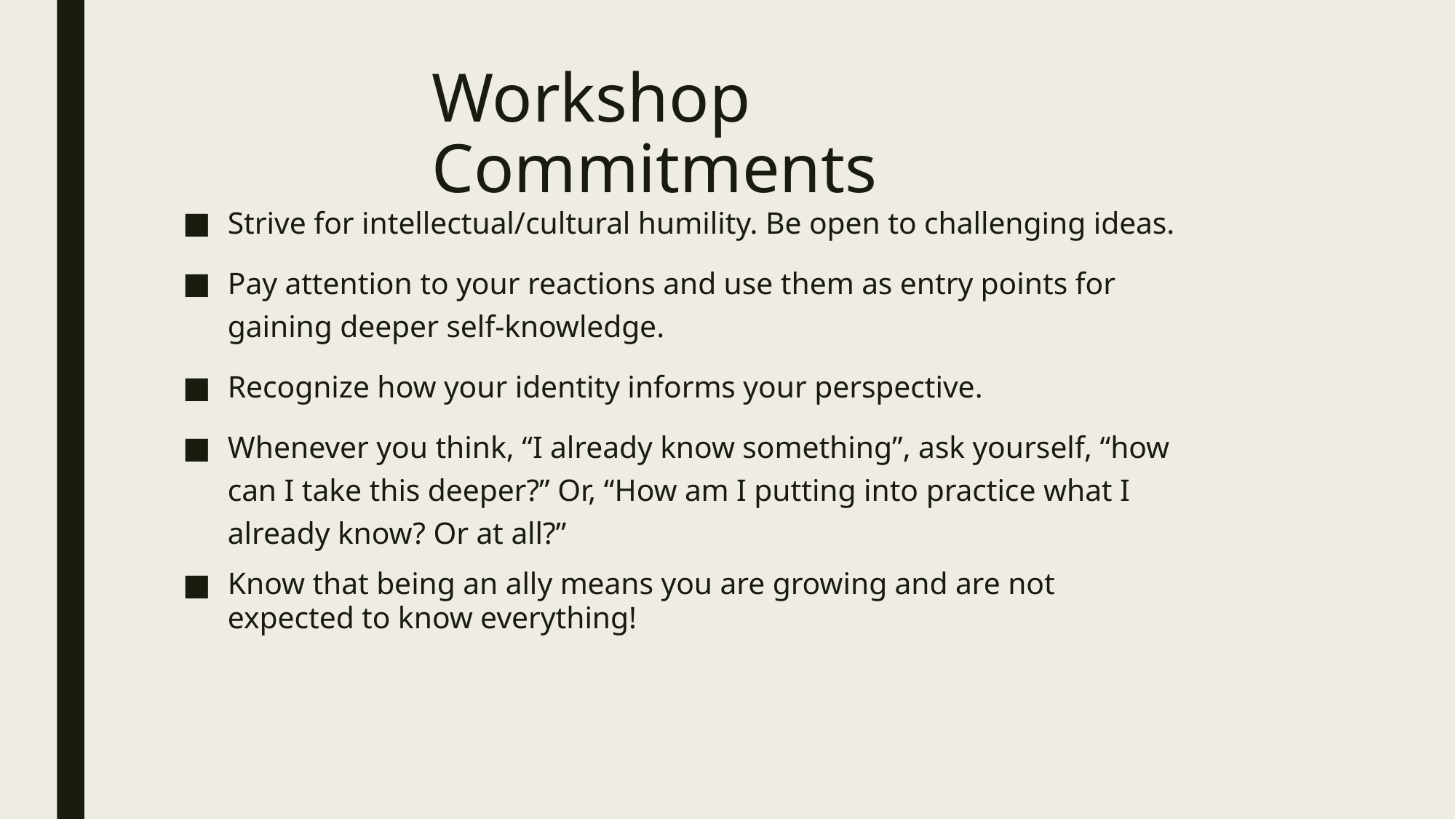

# Workshop Commitments
Strive for intellectual/cultural humility. Be open to challenging ideas.
Pay attention to your reactions and use them as entry points for gaining deeper self-knowledge.
Recognize how your identity informs your perspective.
Whenever you think, “I already know something”, ask yourself, “how can I take this deeper?” Or, “How am I putting into practice what I already know? Or at all?”
Know that being an ally means you are growing and are not expected to know everything!

## Slide 3
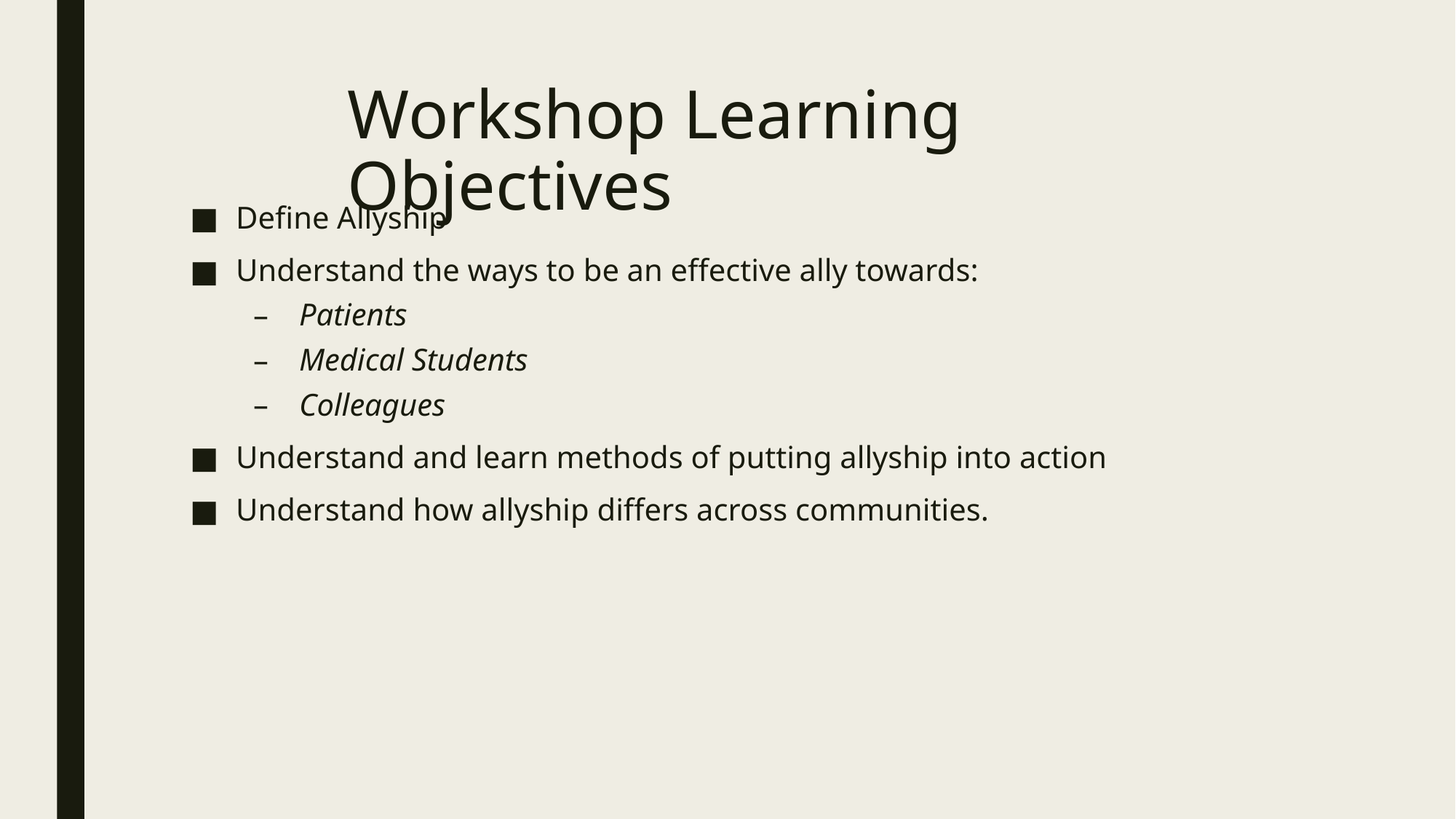

# Workshop Learning Objectives
Define Allyship
Understand the ways to be an effective ally towards:
Patients
Medical Students
Colleagues
Understand and learn methods of putting allyship into action
Understand how allyship differs across communities.

## Slide 4
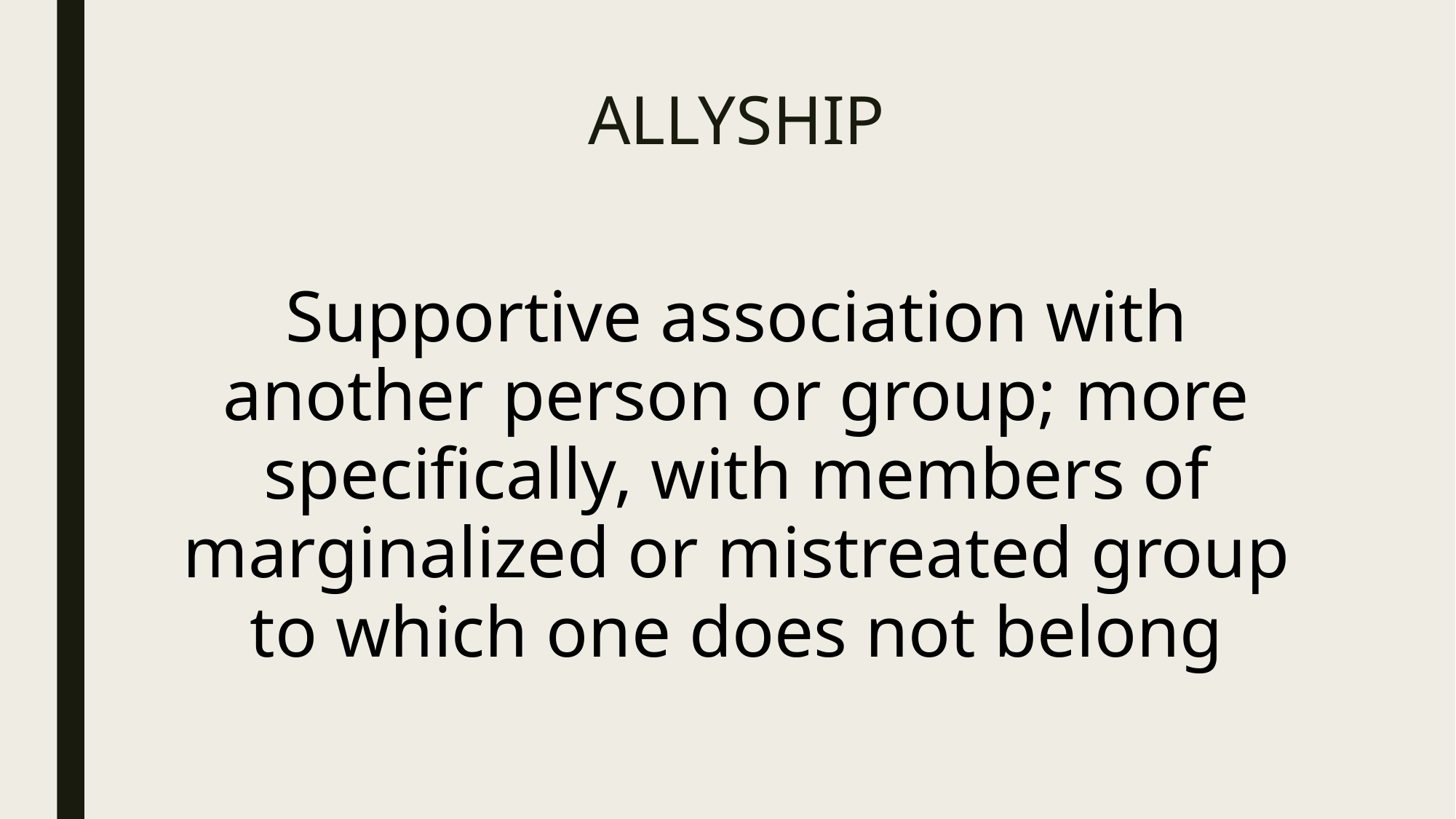

# ALLYSHIP
Supportive association with another person or group; more specifically, with members of marginalized or mistreated group to which one does not belong

## Slide 5
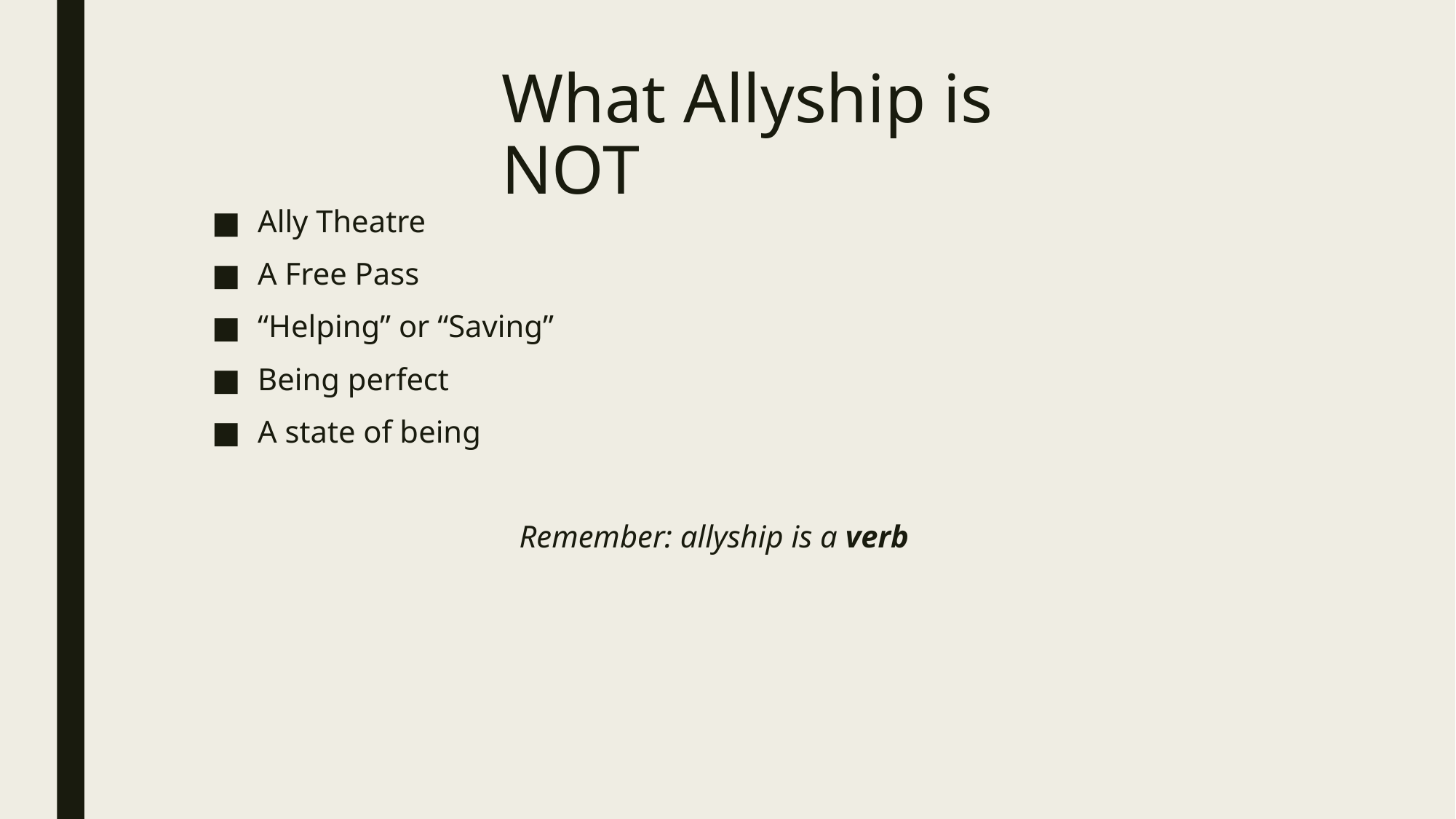

# What Allyship is NOT
Ally Theatre
A Free Pass
“Helping” or “Saving”
Being perfect
A state of being
Remember: allyship is a verb

## Slide 6
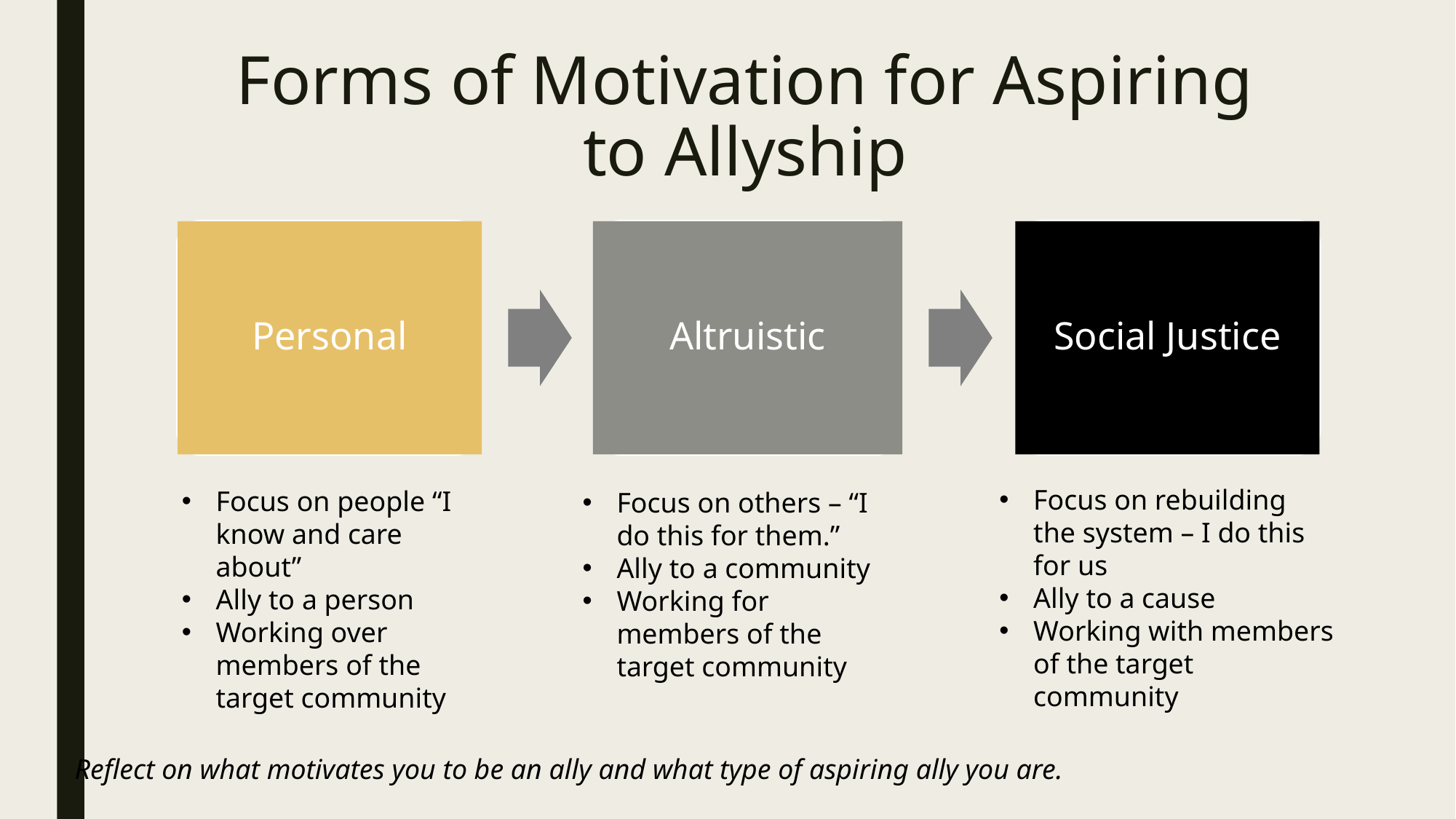

# Forms of Motivation for Aspiring to Allyship
Personal
Altruistic
Social Justice
Focus on rebuilding the system – I do this for us
Ally to a cause
Working with members of the target community
Focus on people “I know and care about”
Ally to a person
Working over members of the target community
Focus on others – “I do this for them.”
Ally to a community
Working for members of the target community
Reflect on what motivates you to be an ally and what type of aspiring ally you are.

## Slide 7
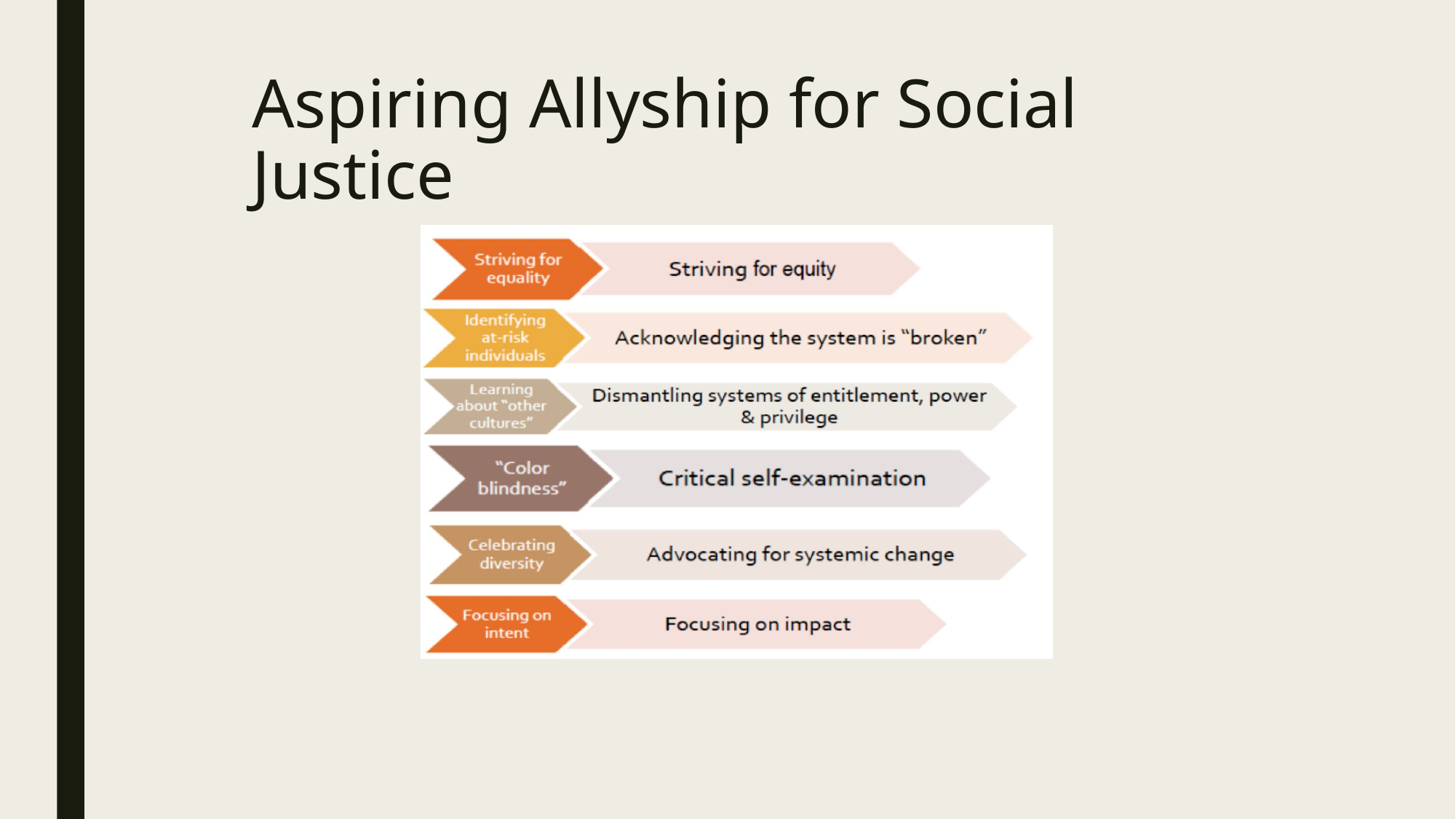

# Aspiring Allyship for Social Justice

## Slide 8
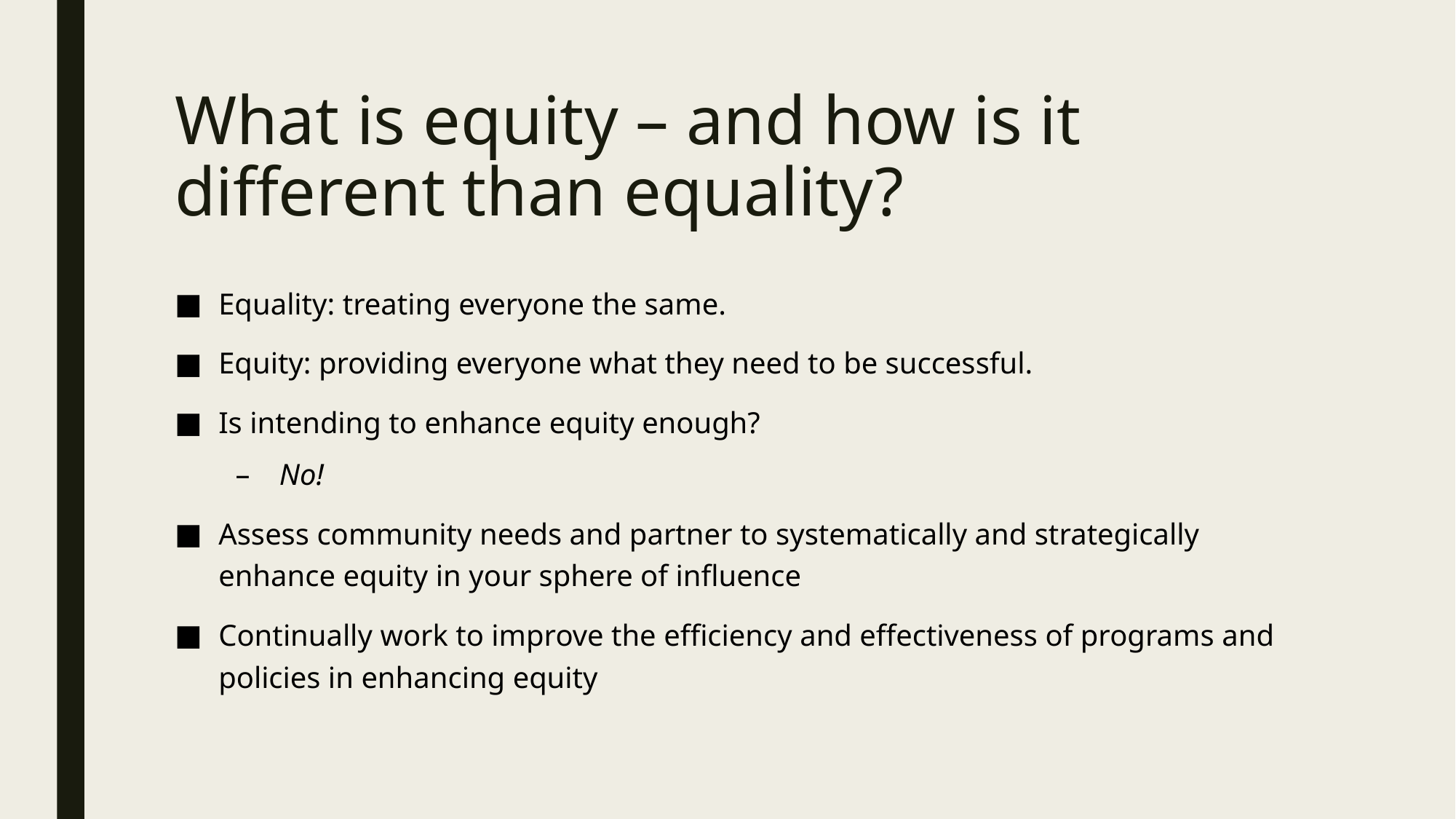

# What is equity – and how is it different than equality?
Equality: treating everyone the same.
Equity: providing everyone what they need to be successful.
Is intending to enhance equity enough?
No!
Assess community needs and partner to systematically and strategically enhance equity in your sphere of influence
Continually work to improve the efficiency and effectiveness of programs and policies in enhancing equity

## Slide 9
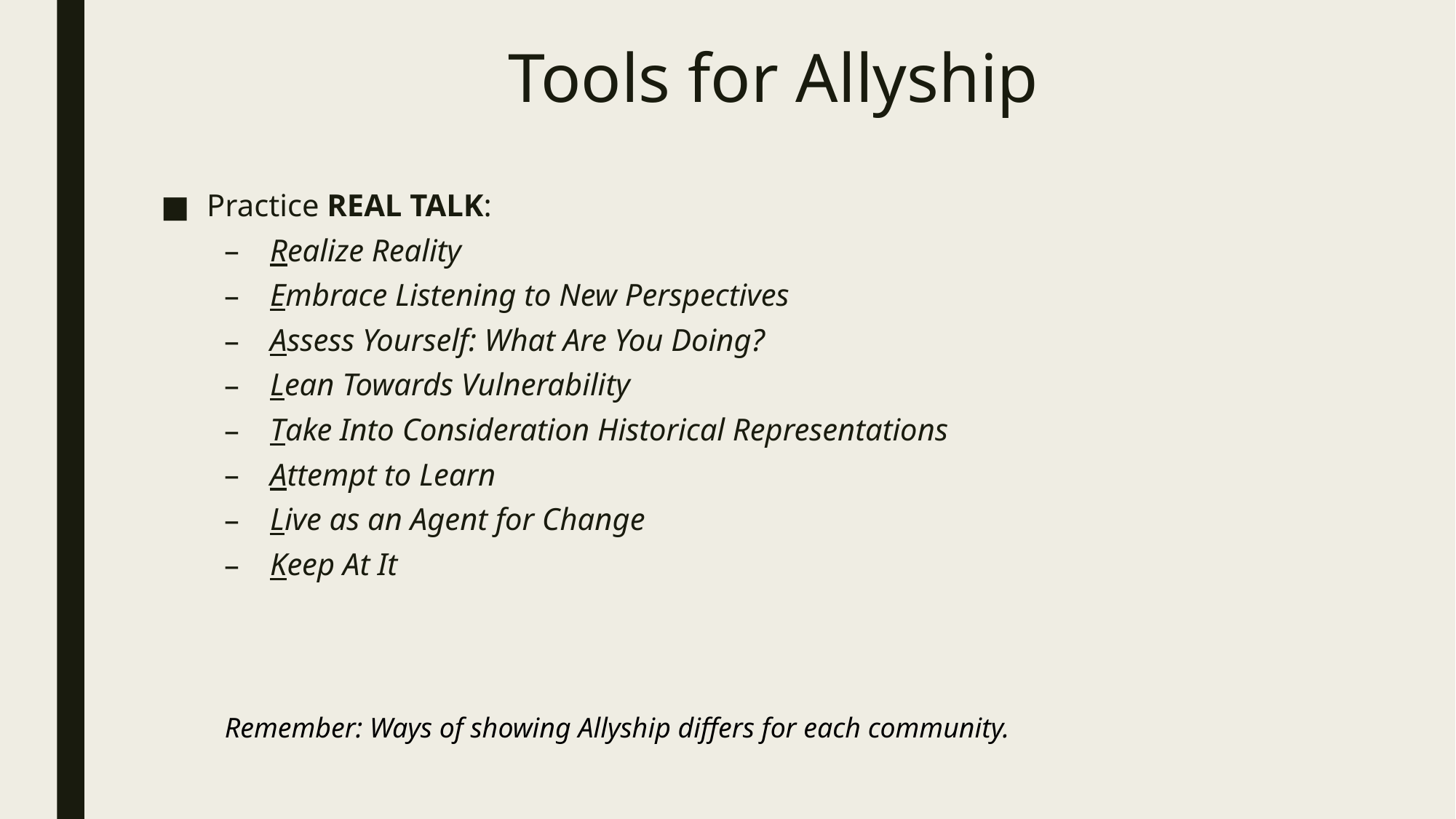

# Tools for Allyship
Practice REAL TALK:
Realize Reality
Embrace Listening to New Perspectives
Assess Yourself: What Are You Doing?
Lean Towards Vulnerability
Take Into Consideration Historical Representations
Attempt to Learn
Live as an Agent for Change
Keep At It
Remember: Ways of showing Allyship differs for each community.

## Slide 10
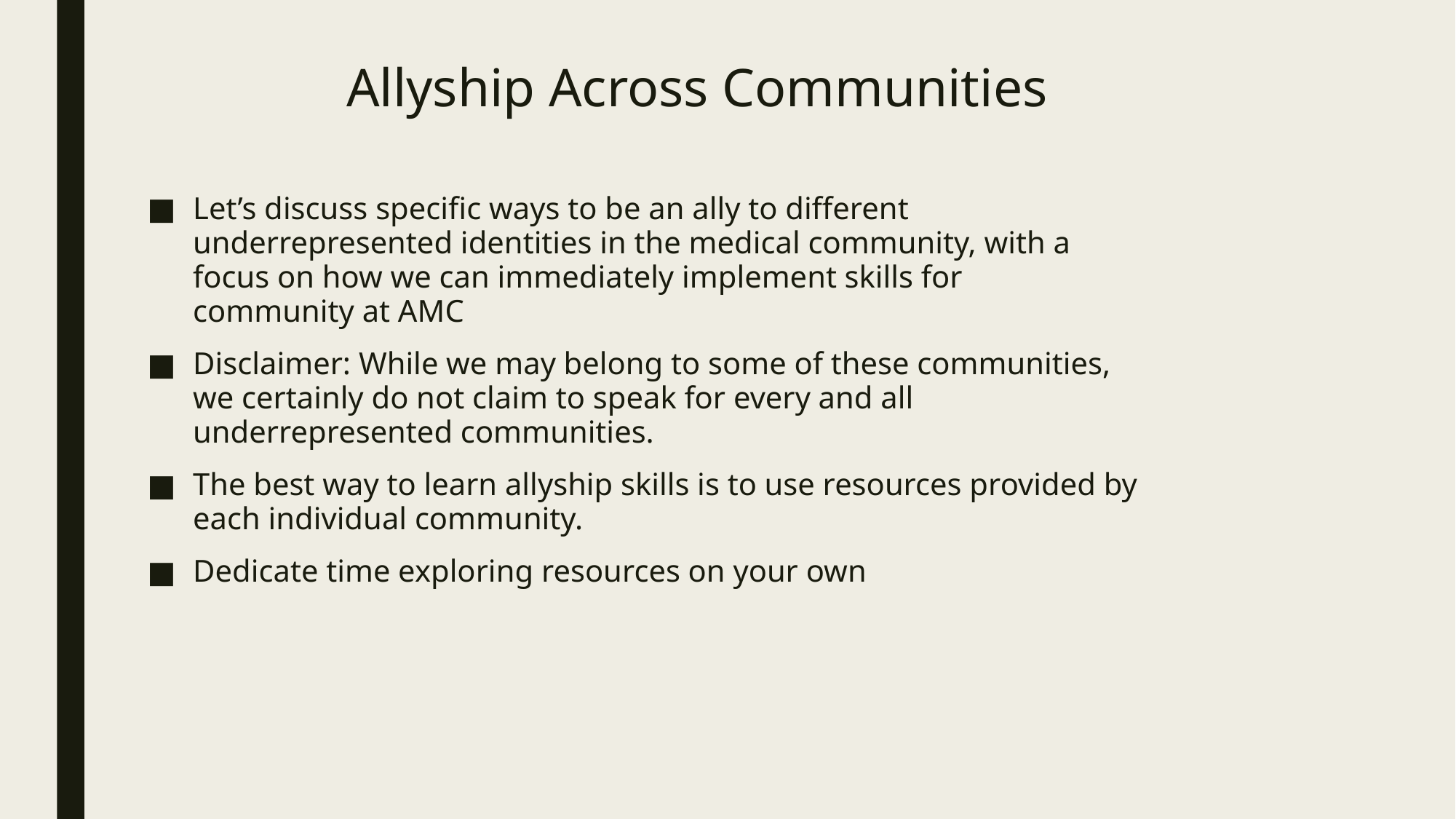

# Allyship Across Communities
Let’s discuss specific ways to be an ally to different underrepresented identities in the medical community, with a focus on how we can immediately implement skills for community at AMC
Disclaimer: While we may belong to some of these communities, we certainly do not claim to speak for every and all underrepresented communities.
The best way to learn allyship skills is to use resources provided by each individual community.
Dedicate time exploring resources on your own

## Slide 11
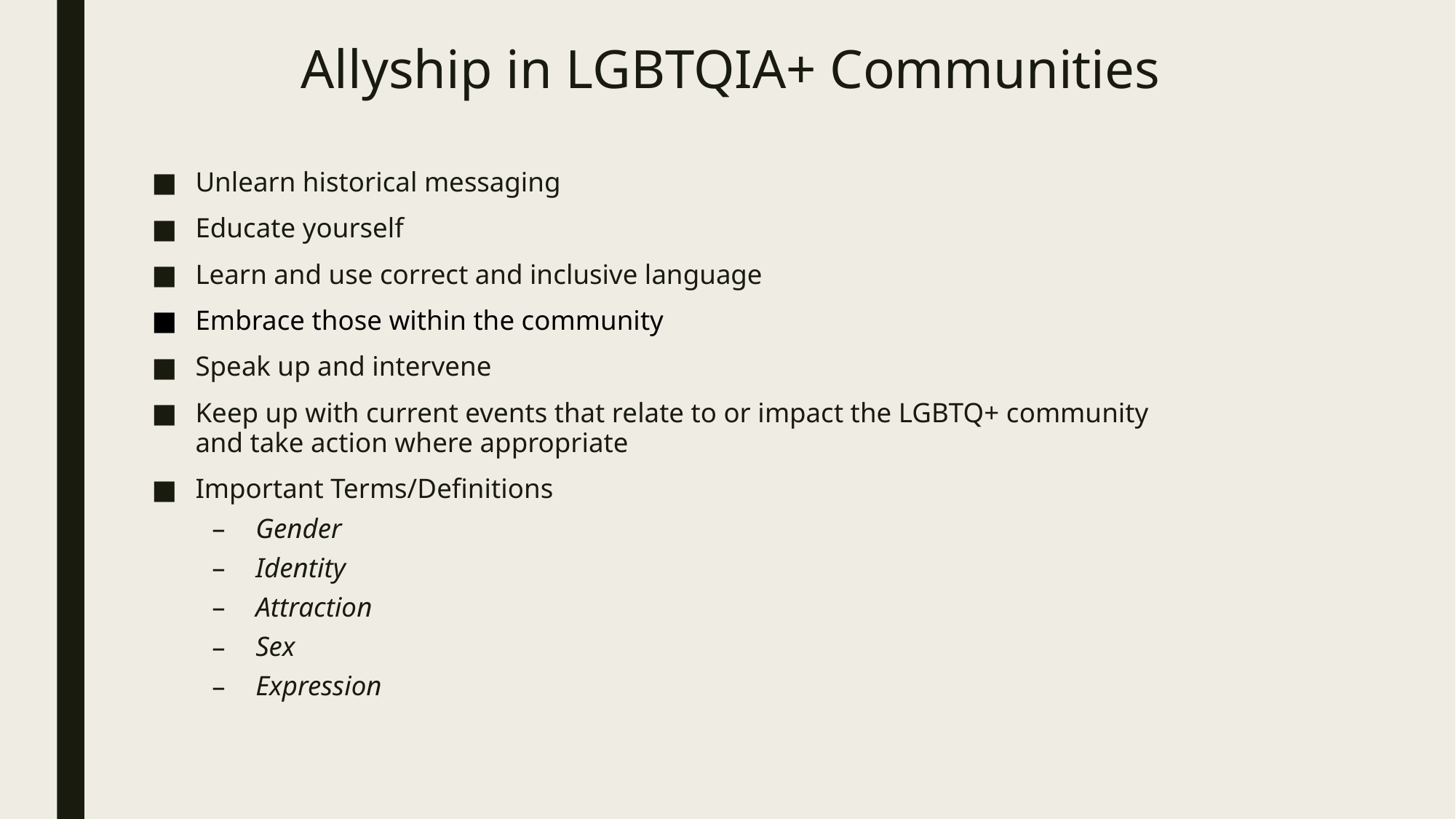

# Allyship in LGBTQIA+ Communities
Unlearn historical messaging
Educate yourself
Learn and use correct and inclusive language
Embrace those within the community
Speak up and intervene
Keep up with current events that relate to or impact the LGBTQ+ community and take action where appropriate
Important Terms/Definitions
Gender
Identity
Attraction
Sex
Expression

## Slide 12
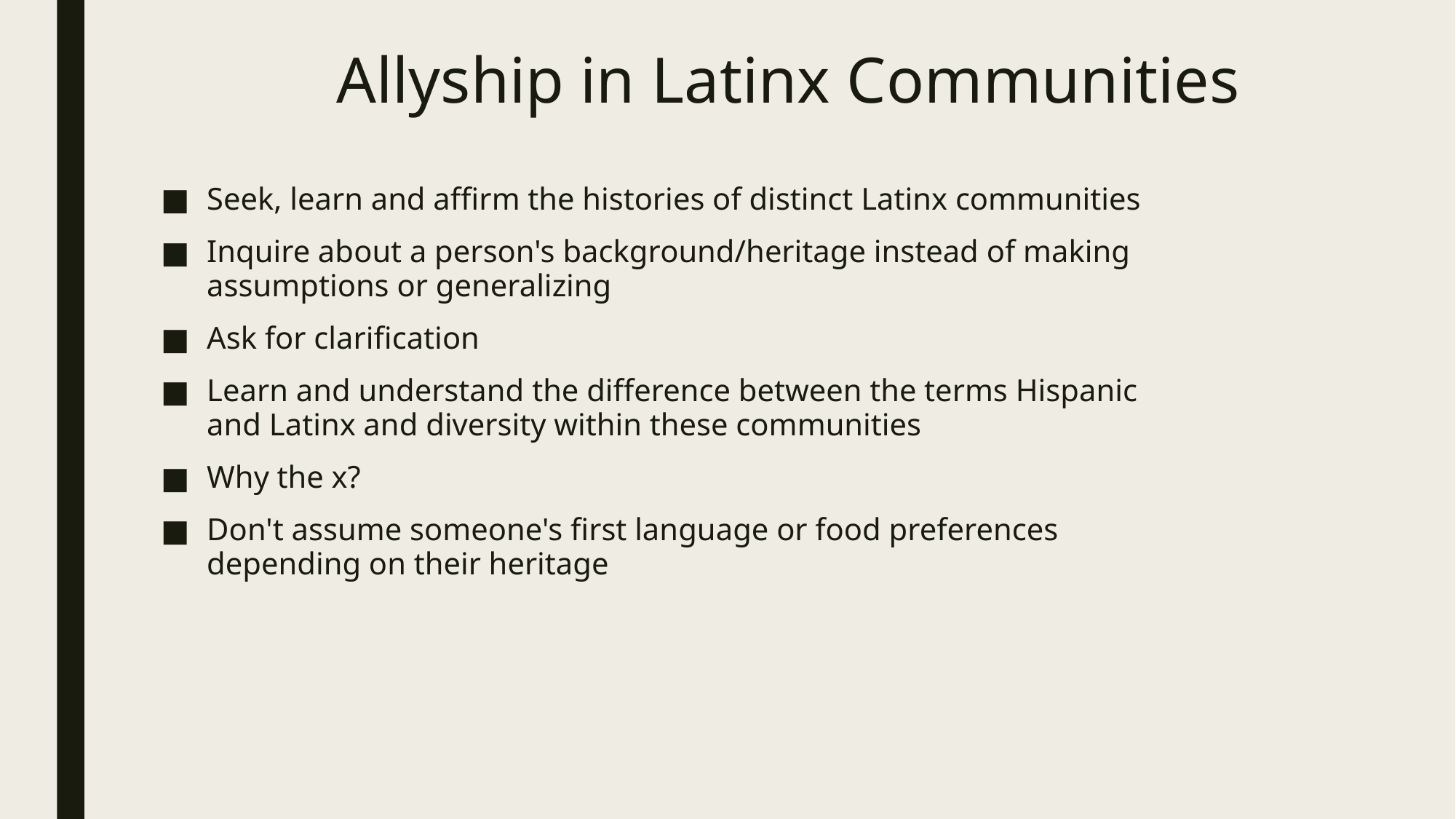

# Allyship in Latinx Communities
Seek, learn and affirm the histories of distinct Latinx communities
Inquire about a person's background/heritage instead of making assumptions or generalizing
Ask for clarification
Learn and understand the difference between the terms Hispanic and Latinx and diversity within these communities
Why the x?
Don't assume someone's first language or food preferences depending on their heritage

## Slide 13
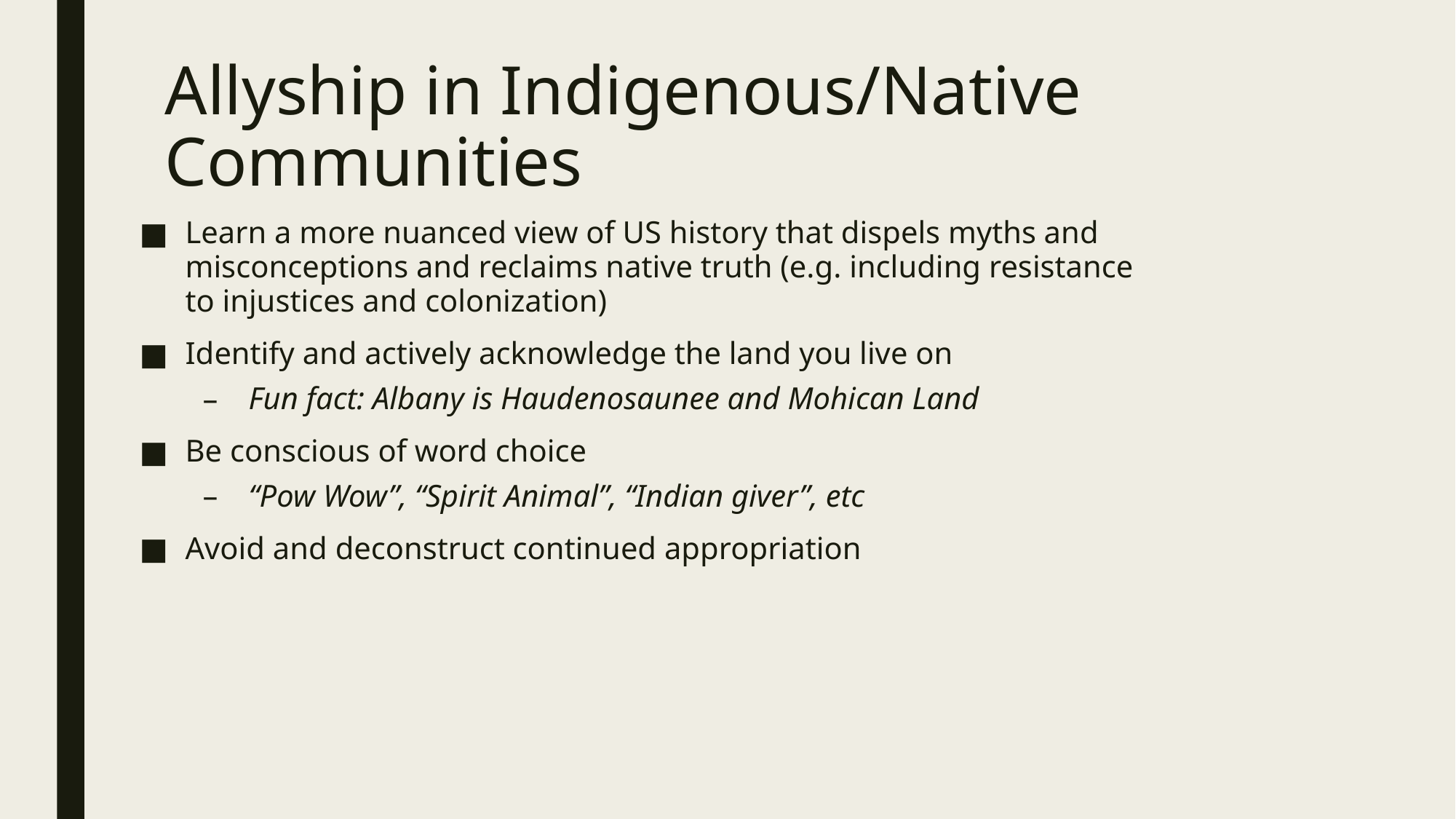

# Allyship in Indigenous/Native Communities
Learn a more nuanced view of US history that dispels myths and misconceptions and reclaims native truth (e.g. including resistance to injustices and colonization)
Identify and actively acknowledge the land you live on
Fun fact: Albany is Haudenosaunee and Mohican Land
Be conscious of word choice
“Pow Wow”, “Spirit Animal”, “Indian giver”, etc
Avoid and deconstruct continued appropriation

## Slide 14
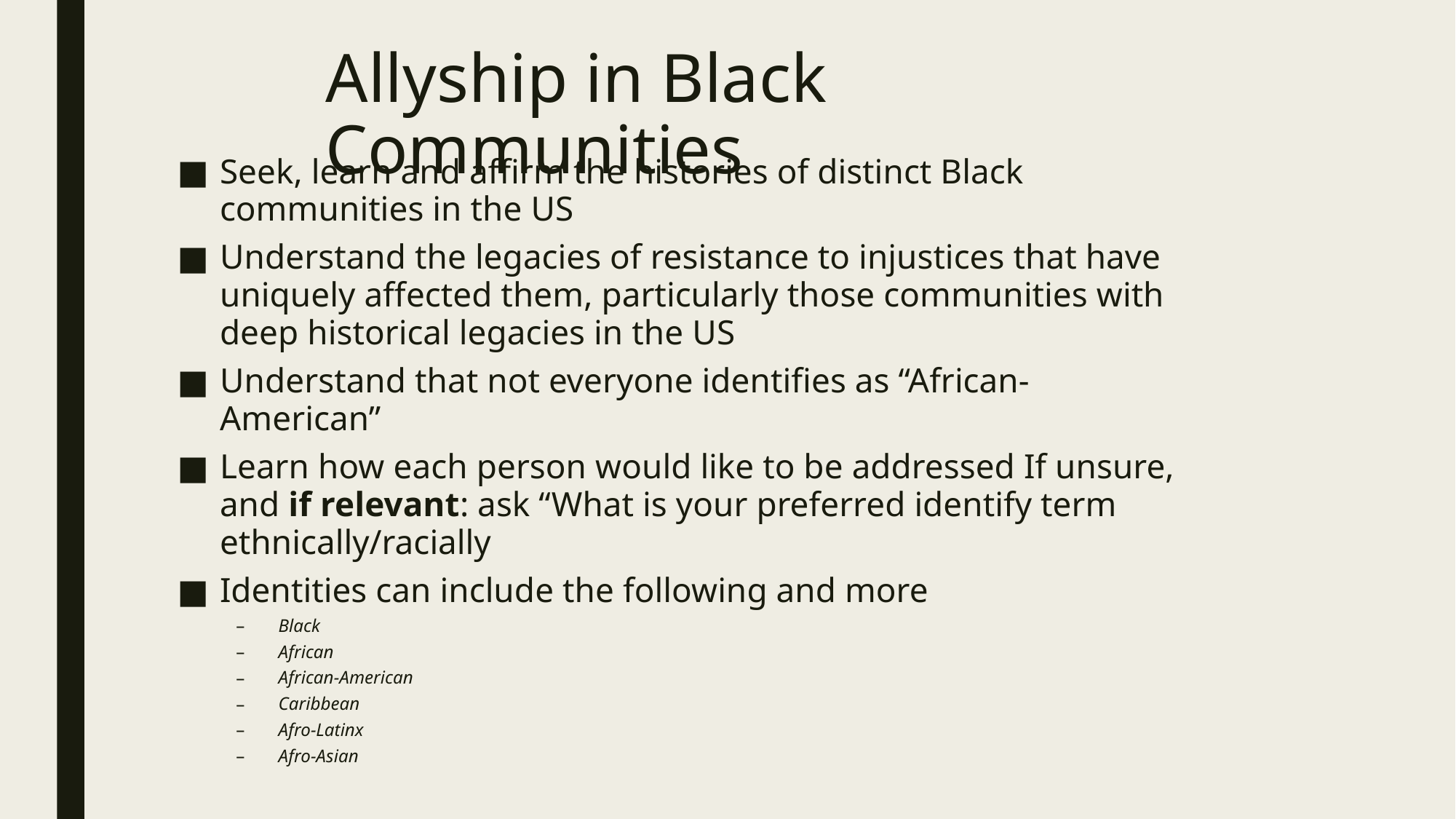

# Allyship in Black Communities
Seek, learn and affirm the histories of distinct Black communities in the US
Understand the legacies of resistance to injustices that have uniquely affected them, particularly those communities with deep historical legacies in the US
Understand that not everyone identifies as “African-American”
Learn how each person would like to be addressed If unsure, and if relevant: ask “What is your preferred identify term ethnically/racially
Identities can include the following and more
Black
African
African-American
Caribbean
Afro-Latinx
Afro-Asian

## Slide 15
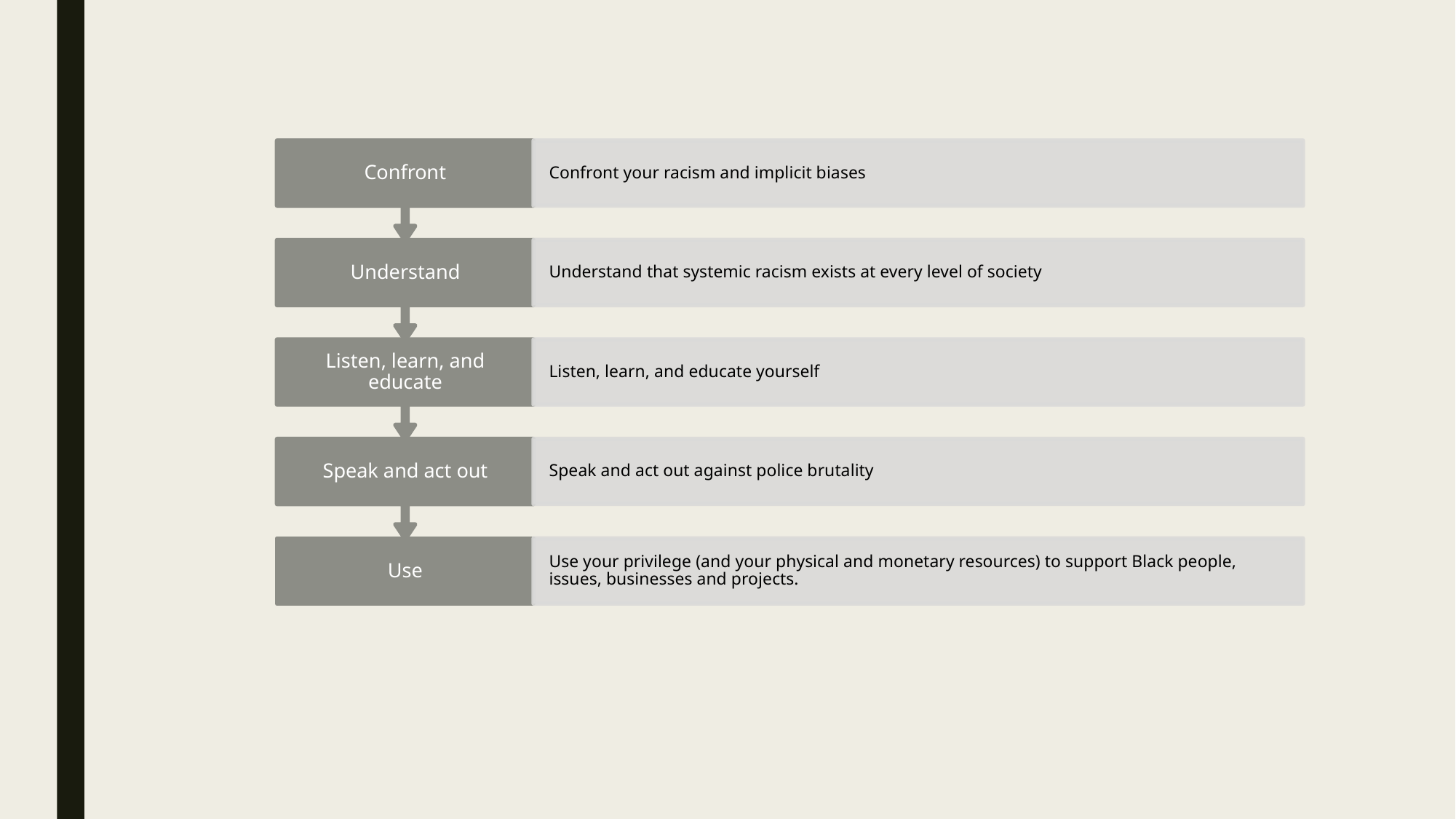

## Slide 16
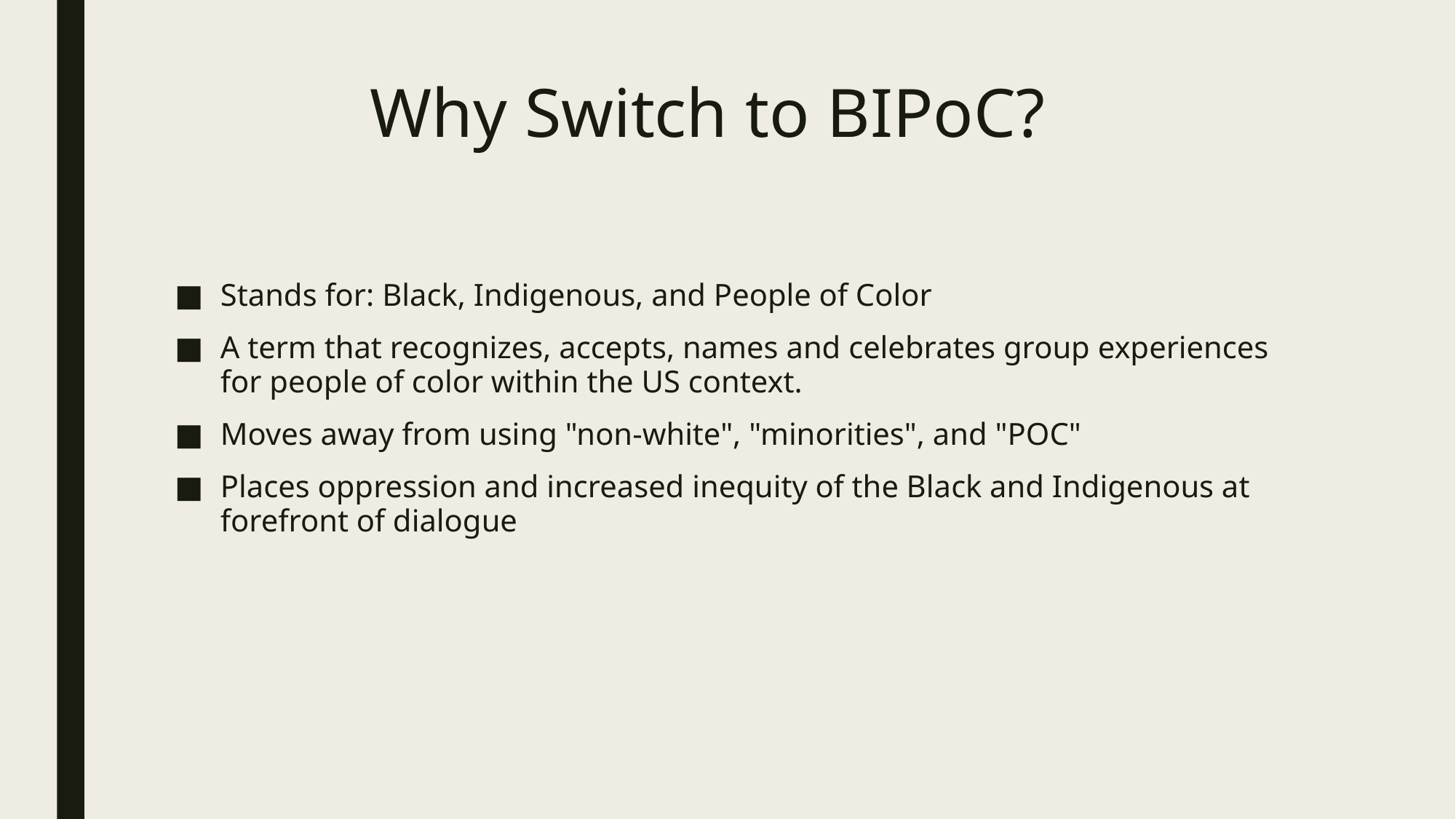

# Why Switch to BIPoC?
Stands for: Black, Indigenous, and People of Color
A term that recognizes, accepts, names and celebrates group experiences for people of color within the US context.
Moves away from using "non-white", "minorities", and "POC"
Places oppression and increased inequity of the Black and Indigenous at forefront of dialogue

## Slide 17
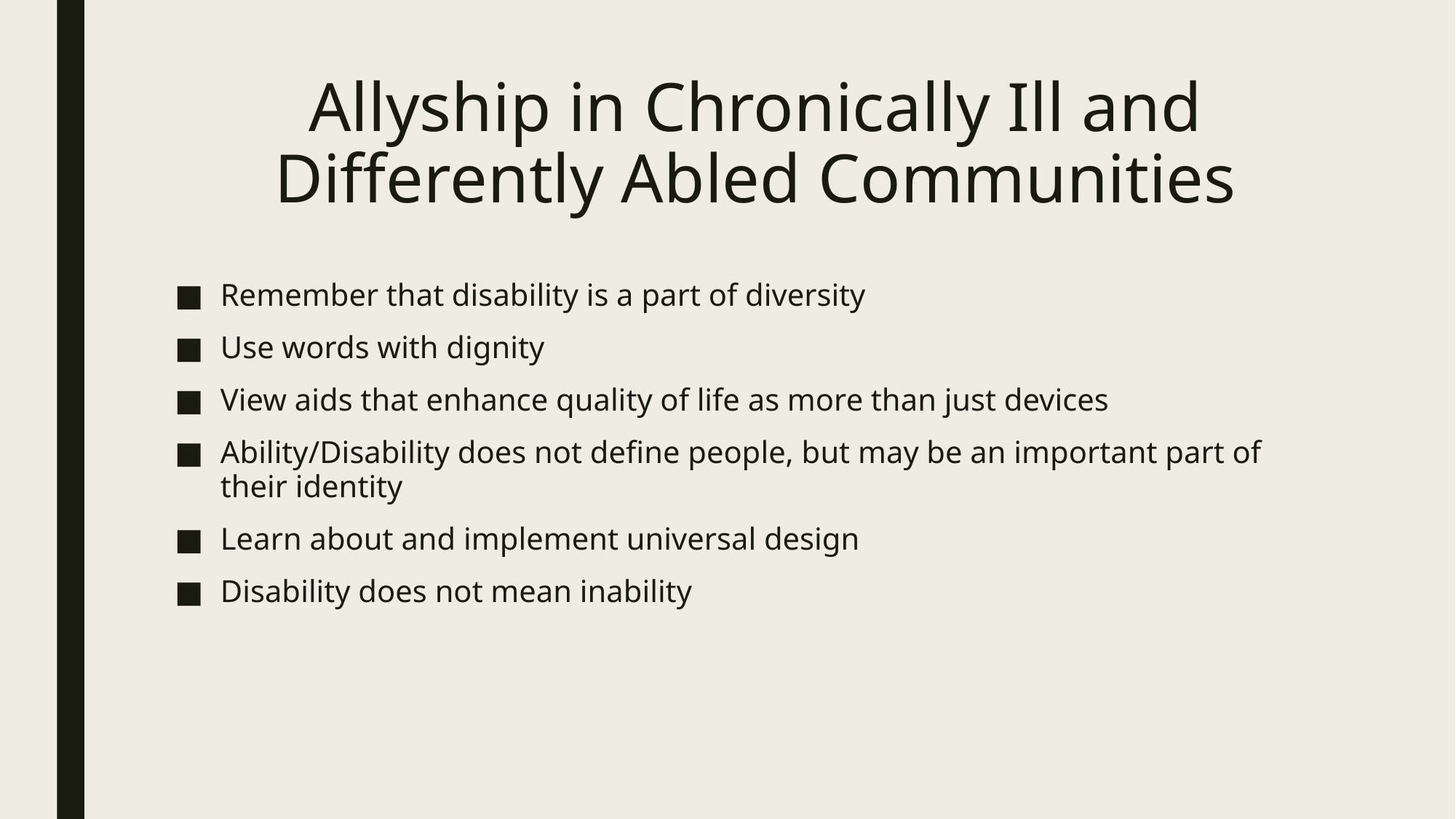

# Allyship in Chronically Ill and Differently Abled Communities
Remember that disability is a part of diversity
Use words with dignity
View aids that enhance quality of life as more than just devices
Ability/Disability does not define people, but may be an important part of their identity
Learn about and implement universal design
Disability does not mean inability

## Slide 18
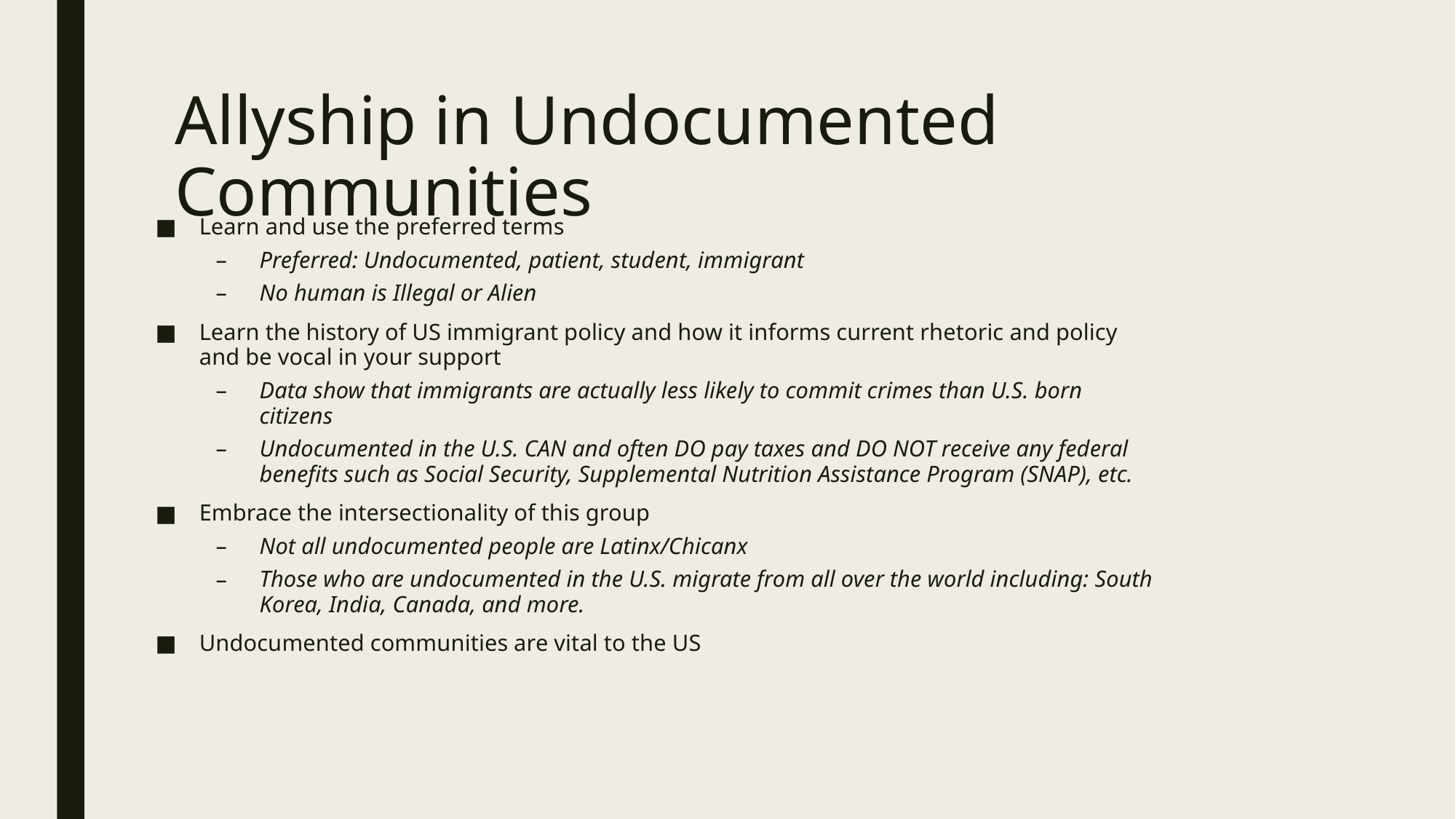

# Allyship in Undocumented Communities
Learn and use the preferred terms
Preferred: Undocumented, patient, student, immigrant
No human is Illegal or Alien
Learn the history of US immigrant policy and how it informs current rhetoric and policy and be vocal in your support
Data show that immigrants are actually less likely to commit crimes than U.S. born citizens
Undocumented in the U.S. CAN and often DO pay taxes and DO NOT receive any federal benefits such as Social Security, Supplemental Nutrition Assistance Program (SNAP), etc.
Embrace the intersectionality of this group
Not all undocumented people are Latinx/Chicanx
Those who are undocumented in the U.S. migrate from all over the world including: South Korea, India, Canada, and more.
Undocumented communities are vital to the US

## Slide 19
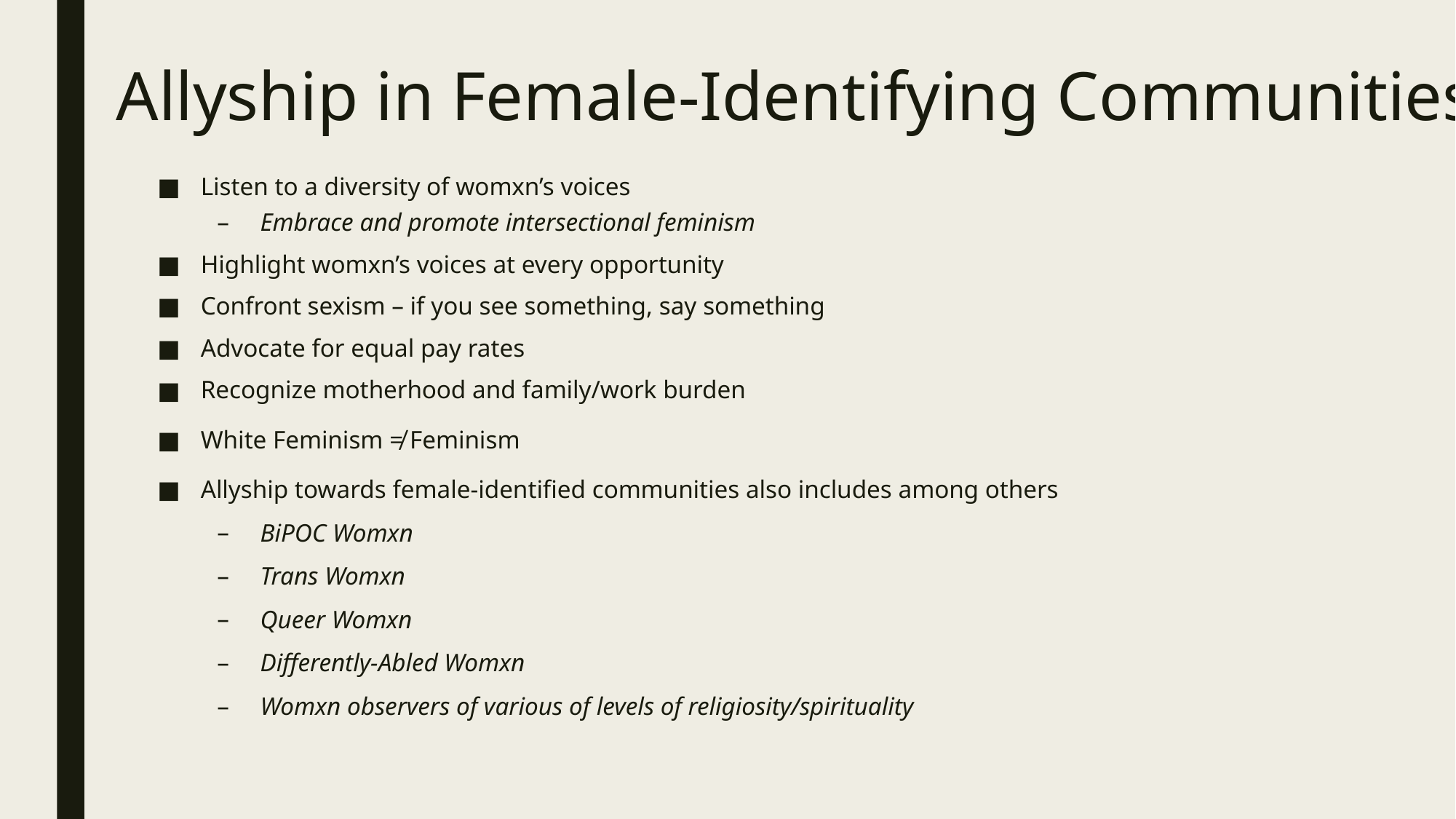

# Allyship in Female-Identifying Communities
Listen to a diversity of womxn’s voices
Embrace and promote intersectional feminism
Highlight womxn’s voices at every opportunity
Confront sexism – if you see something, say something
Advocate for equal pay rates
Recognize motherhood and family/work burden
White Feminism ≠ Feminism
Allyship towards female-identified communities also includes among others
BiPOC Womxn
Trans Womxn
Queer Womxn
Differently-Abled Womxn
Womxn observers of various of levels of religiosity/spirituality

## Slide 20
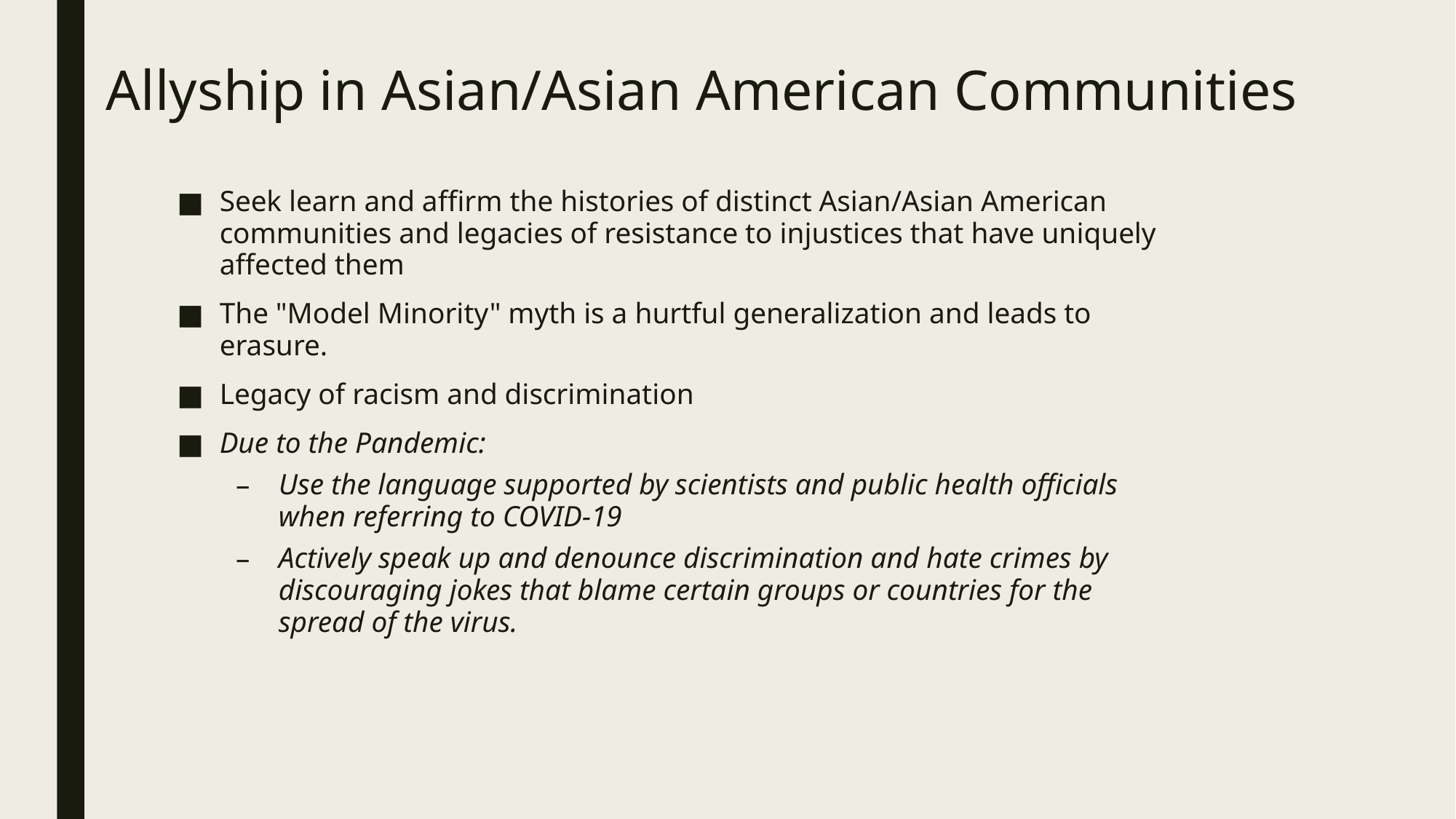

# Allyship in Asian/Asian American Communities
Seek learn and affirm the histories of distinct Asian/Asian American communities and legacies of resistance to injustices that have uniquely affected them
The "Model Minority" myth is a hurtful generalization and leads to erasure.
Legacy of racism and discrimination
Due to the Pandemic:
Use the language supported by scientists and public health officials when referring to COVID-19
Actively speak up and denounce discrimination and hate crimes by discouraging jokes that blame certain groups or countries for the spread of the virus.

## Slide 21
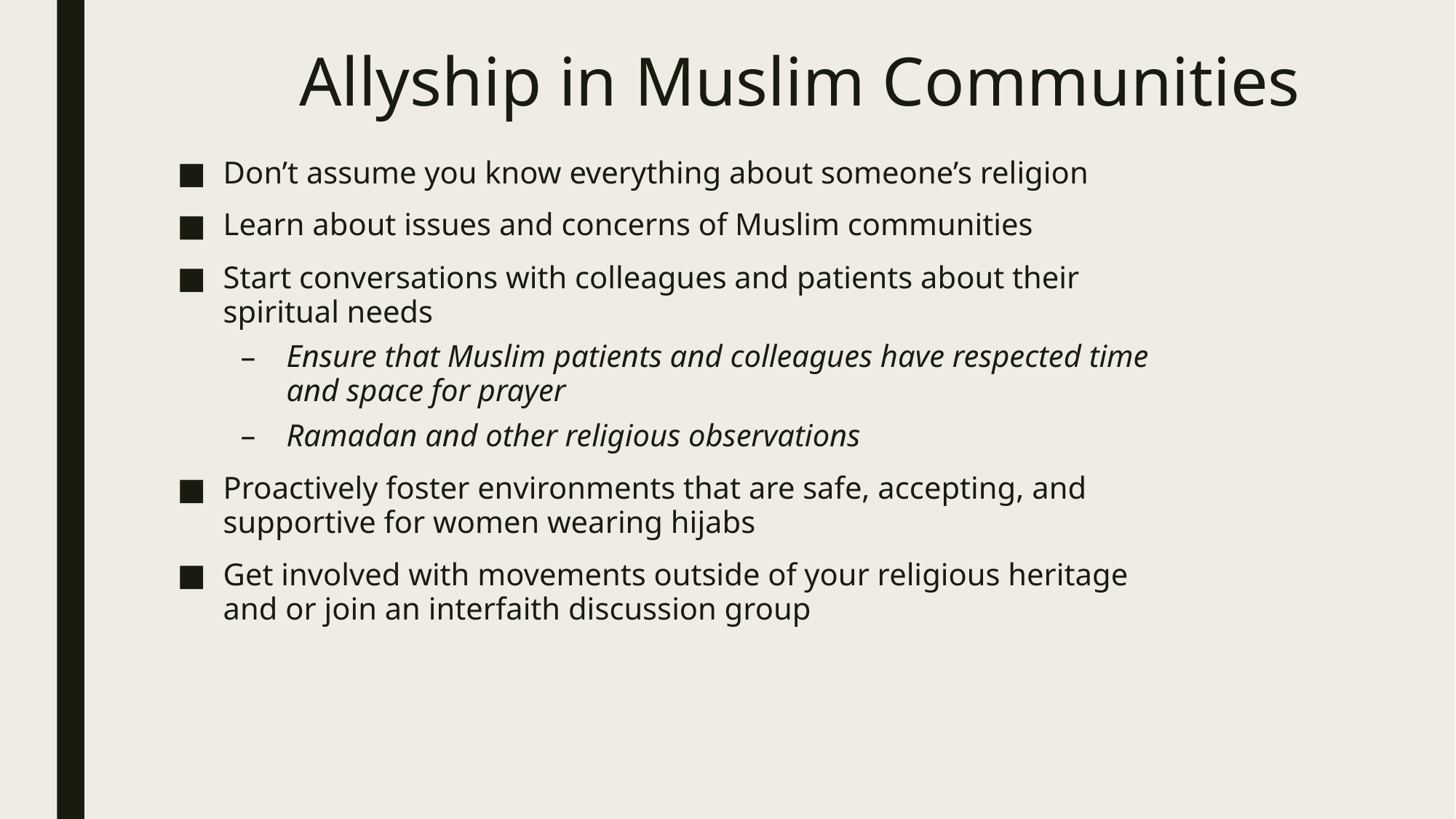

# Allyship in Muslim Communities
Don’t assume you know everything about someone’s religion
Learn about issues and concerns of Muslim communities
Start conversations with colleagues and patients about their spiritual needs
Ensure that Muslim patients and colleagues have respected time and space for prayer
Ramadan and other religious observations
Proactively foster environments that are safe, accepting, and supportive for women wearing hijabs
Get involved with movements outside of your religious heritage and or join an interfaith discussion group

## Slide 22
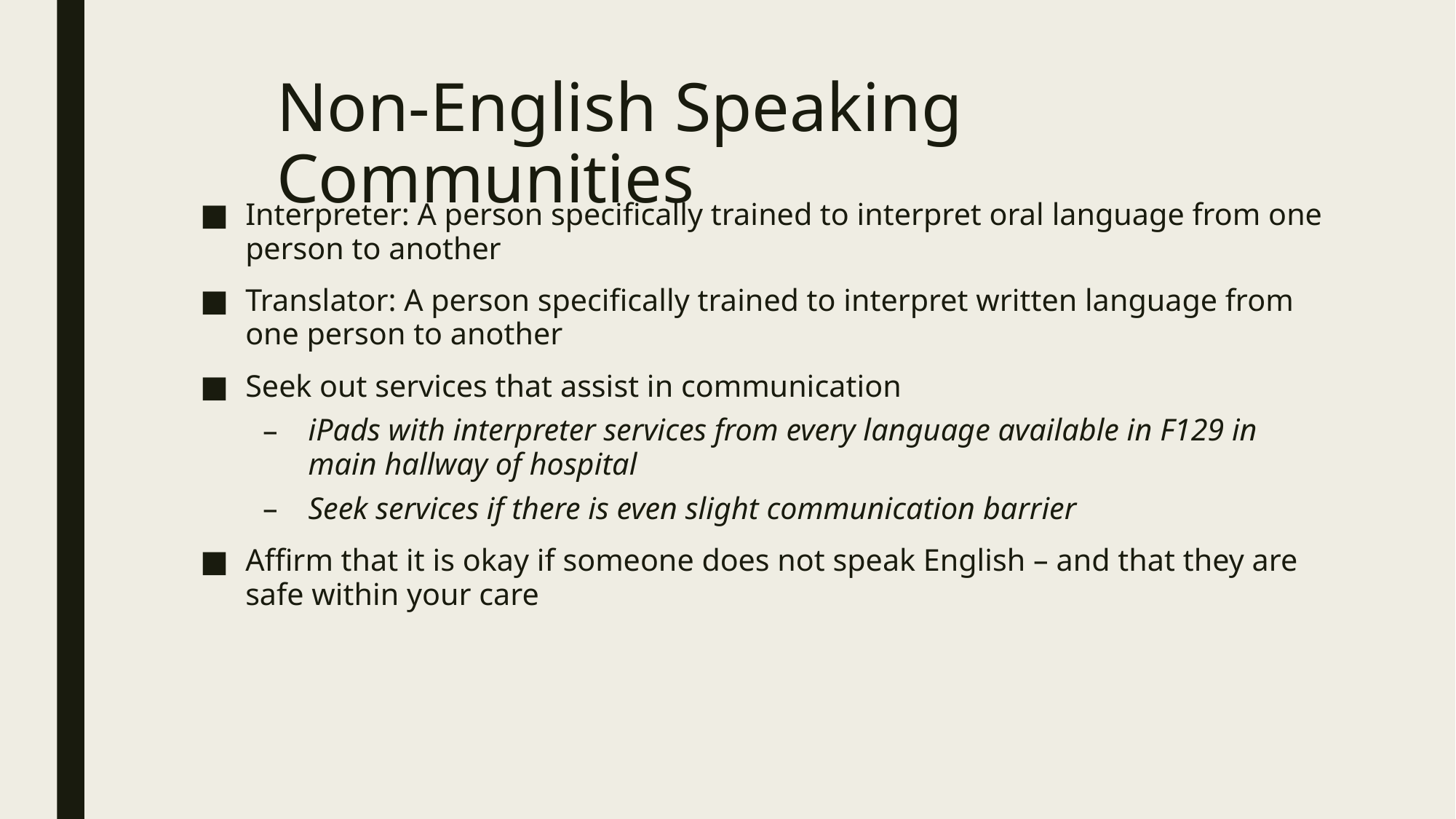

# Non-English Speaking Communities
Interpreter: A person specifically trained to interpret oral language from one person to another
Translator: A person specifically trained to interpret written language from one person to another
Seek out services that assist in communication
iPads with interpreter services from every language available in F129 in main hallway of hospital
Seek services if there is even slight communication barrier
Affirm that it is okay if someone does not speak English – and that they are safe within your care

## Slide 23
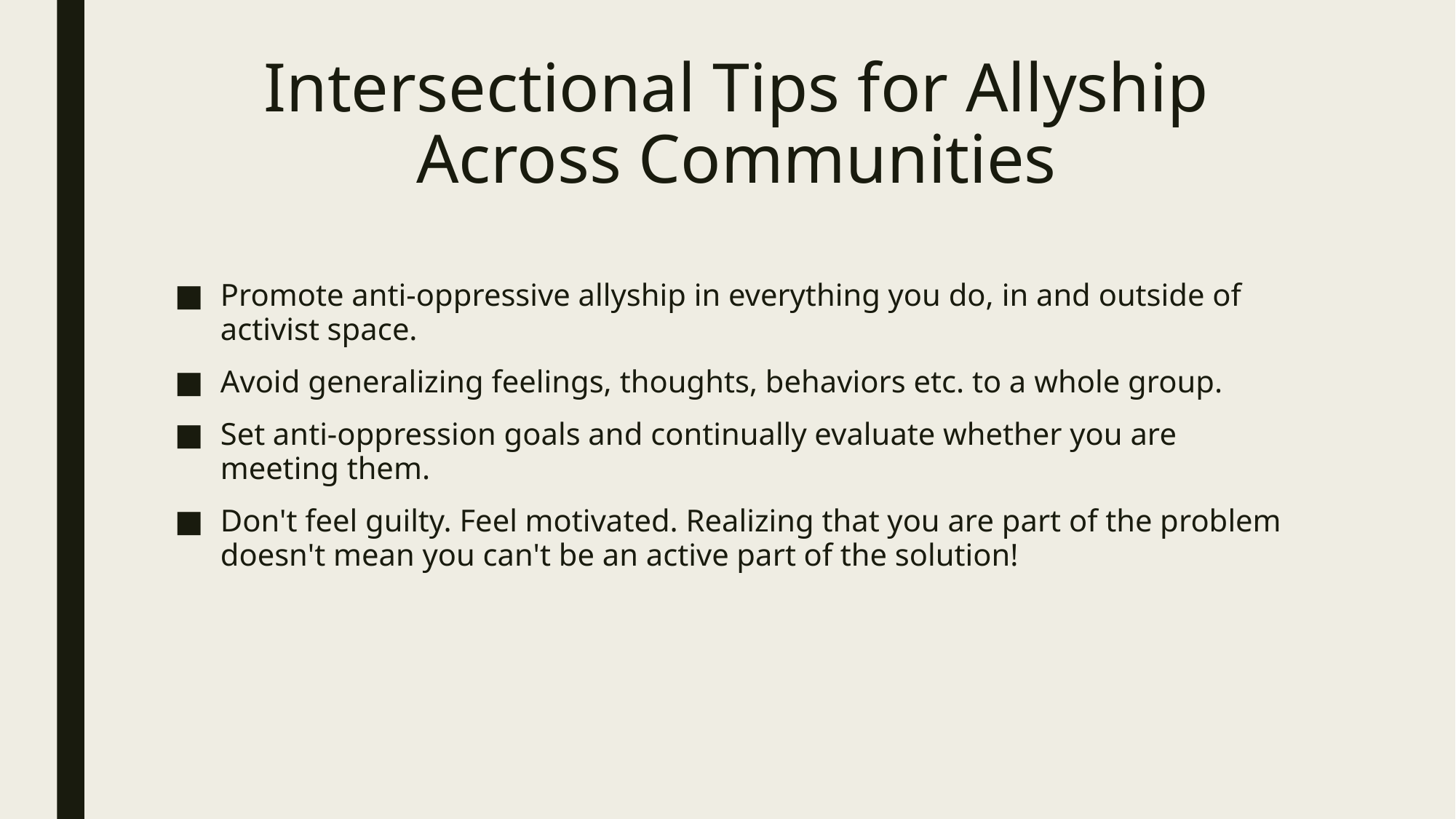

# Intersectional Tips for Allyship Across Communities
Promote anti-oppressive allyship in everything you do, in and outside of activist space.
Avoid generalizing feelings, thoughts, behaviors etc. to a whole group.
Set anti-oppression goals and continually evaluate whether you are meeting them.
Don't feel guilty. Feel motivated. Realizing that you are part of the problem doesn't mean you can't be an active part of the solution!

## Slide 24
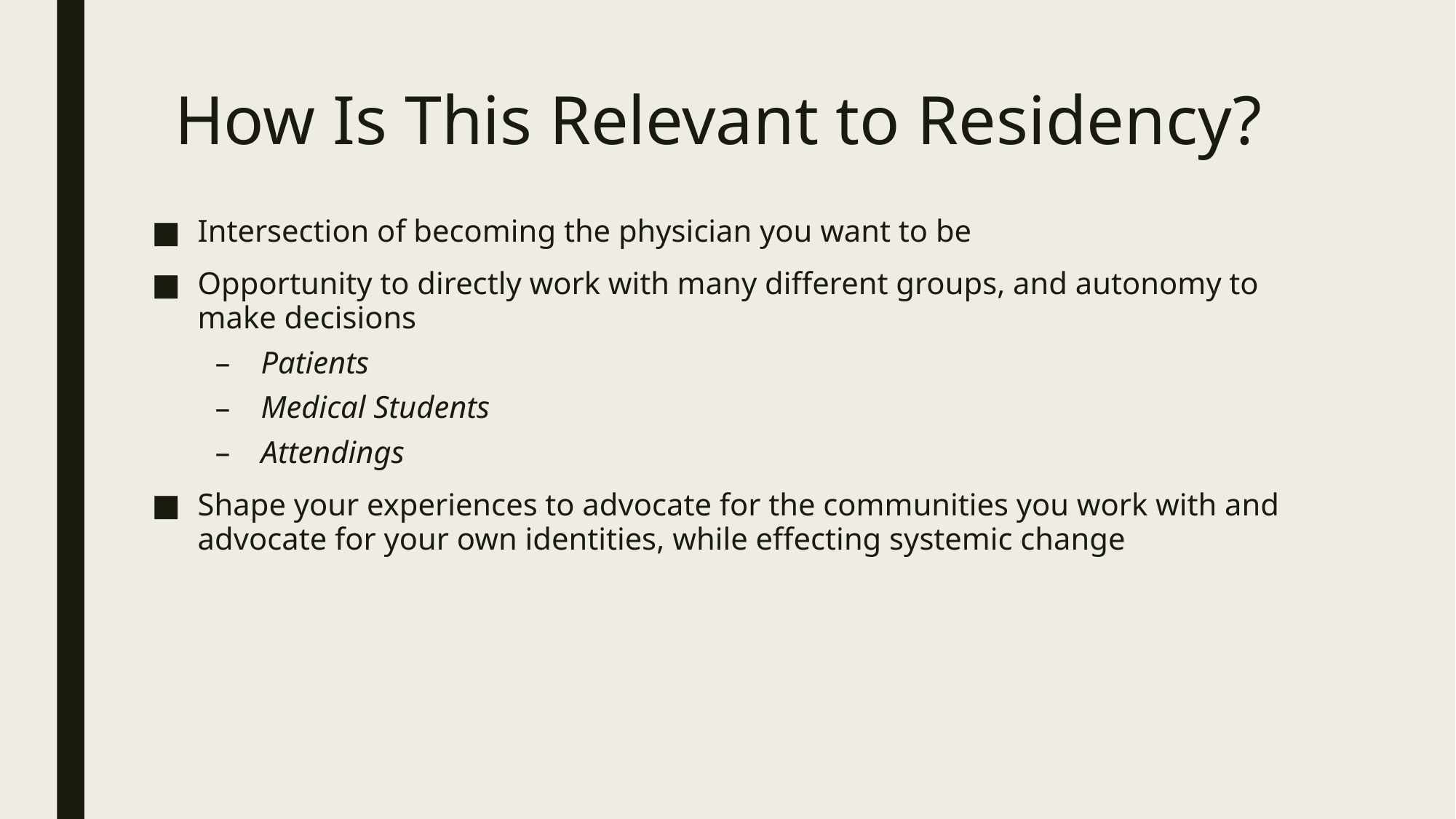

# How Is This Relevant to Residency?
Intersection of becoming the physician you want to be
Opportunity to directly work with many different groups, and autonomy to make decisions
Patients
Medical Students
Attendings
Shape your experiences to advocate for the communities you work with and advocate for your own identities, while effecting systemic change

## Slide 25
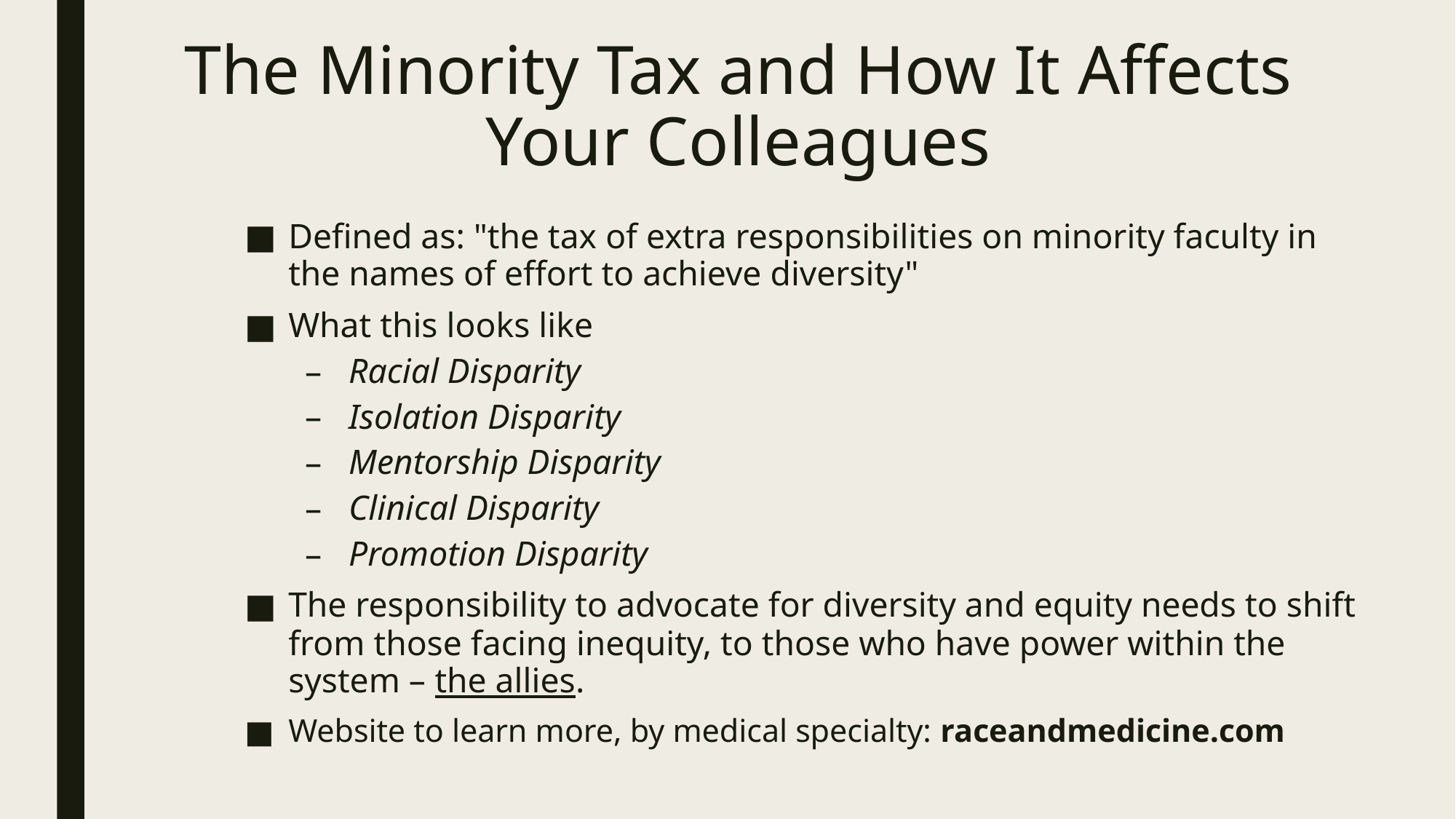

# The Minority Tax and How It Affects Your Colleagues
Defined as: "the tax of extra responsibilities on minority faculty in the names of effort to achieve diversity"
What this looks like
Racial Disparity
Isolation Disparity
Mentorship Disparity
Clinical Disparity
Promotion Disparity
The responsibility to advocate for diversity and equity needs to shift from those facing inequity, to those who have power within the system – the allies.
Website to learn more, by medical specialty: raceandmedicine.com

## Slide 26
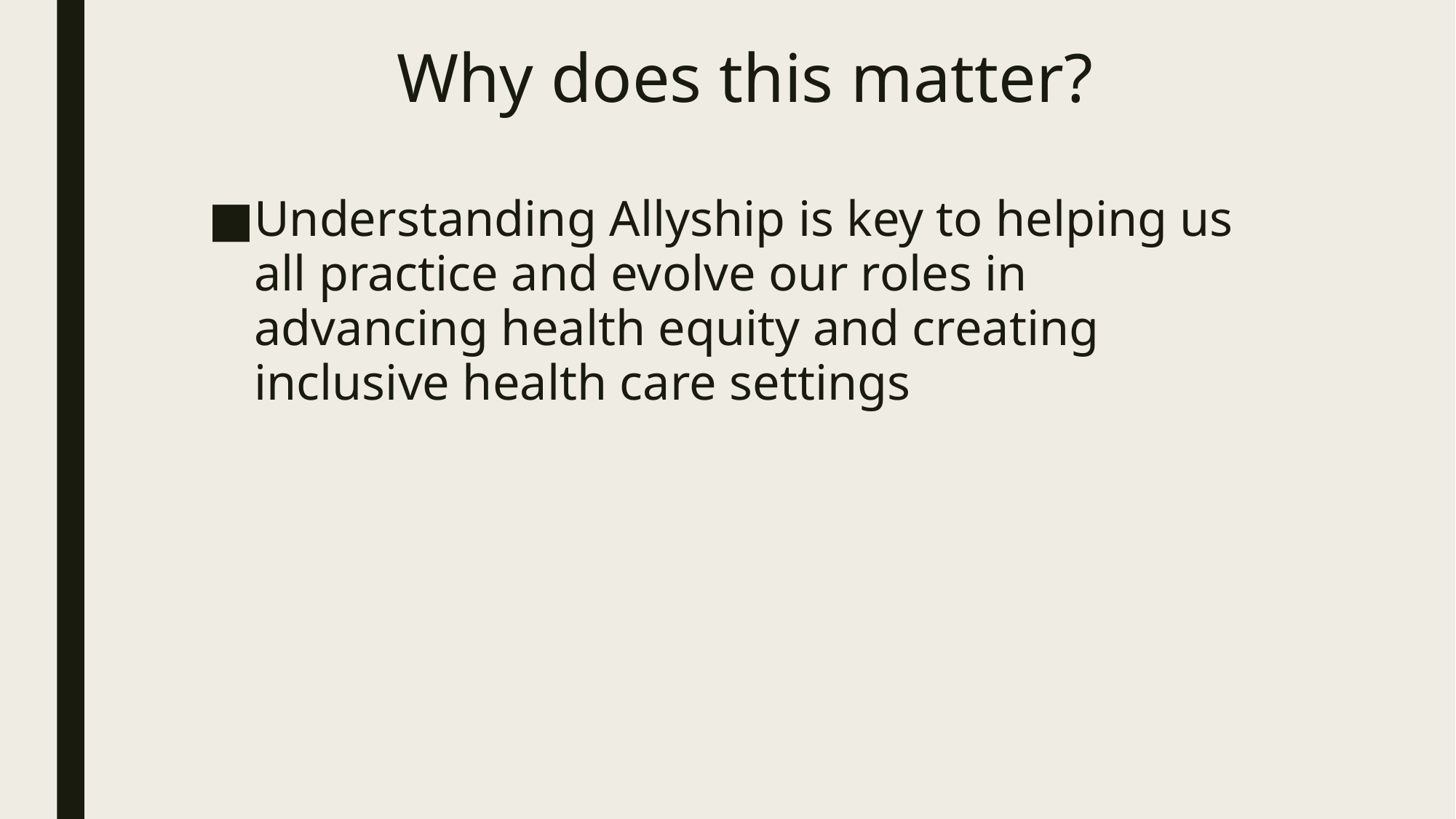

# Why does this matter?
Understanding Allyship is key to helping us all practice and evolve our roles in advancing health equity and creating inclusive health care settings

## Slide 27
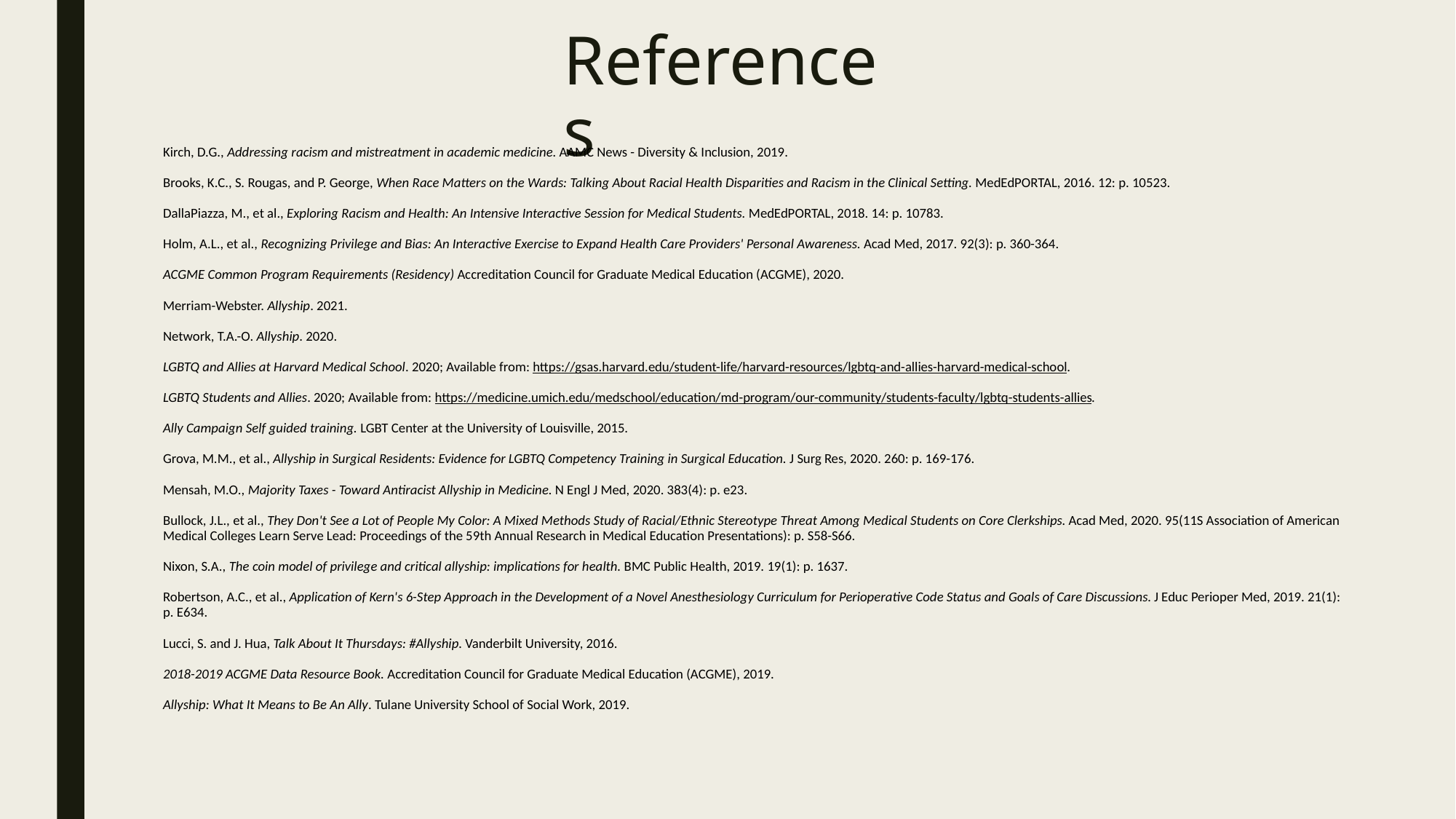

# References
Kirch, D.G., Addressing racism and mistreatment in academic medicine. AAMC News - Diversity & Inclusion, 2019.
Brooks, K.C., S. Rougas, and P. George, When Race Matters on the Wards: Talking About Racial Health Disparities and Racism in the Clinical Setting. MedEdPORTAL, 2016. 12: p. 10523.
DallaPiazza, M., et al., Exploring Racism and Health: An Intensive Interactive Session for Medical Students. MedEdPORTAL, 2018. 14: p. 10783.
Holm, A.L., et al., Recognizing Privilege and Bias: An Interactive Exercise to Expand Health Care Providers' Personal Awareness. Acad Med, 2017. 92(3): p. 360-364.
ACGME Common Program Requirements (Residency) Accreditation Council for Graduate Medical Education (ACGME), 2020.
Merriam-Webster. Allyship. 2021.
Network, T.A.-O. Allyship. 2020.
LGBTQ and Allies at Harvard Medical School. 2020; Available from: https://gsas.harvard.edu/student-life/harvard-resources/lgbtq-and-allies-harvard-medical-school.
LGBTQ Students and Allies. 2020; Available from: https://medicine.umich.edu/medschool/education/md-program/our-community/students-faculty/lgbtq-students-allies.
Ally Campaign Self guided training. LGBT Center at the University of Louisville, 2015.
Grova, M.M., et al., Allyship in Surgical Residents: Evidence for LGBTQ Competency Training in Surgical Education. J Surg Res, 2020. 260: p. 169-176.
Mensah, M.O., Majority Taxes - Toward Antiracist Allyship in Medicine. N Engl J Med, 2020. 383(4): p. e23.
Bullock, J.L., et al., They Don't See a Lot of People My Color: A Mixed Methods Study of Racial/Ethnic Stereotype Threat Among Medical Students on Core Clerkships. Acad Med, 2020. 95(11S Association of American Medical Colleges Learn Serve Lead: Proceedings of the 59th Annual Research in Medical Education Presentations): p. S58-S66.
Nixon, S.A., The coin model of privilege and critical allyship: implications for health. BMC Public Health, 2019. 19(1): p. 1637.
Robertson, A.C., et al., Application of Kern's 6-Step Approach in the Development of a Novel Anesthesiology Curriculum for Perioperative Code Status and Goals of Care Discussions. J Educ Perioper Med, 2019. 21(1): p. E634.
Lucci, S. and J. Hua, Talk About It Thursdays: #Allyship. Vanderbilt University, 2016.
2018-2019 ACGME Data Resource Book. Accreditation Council for Graduate Medical Education (ACGME), 2019.
Allyship: What It Means to Be An Ally. Tulane University School of Social Work, 2019.
