# Supplementary material for: Allyship in Residency: An Introductory Module on Medical Allyship for Graduate Medical Trainees
Source: MedEdPORTAL. 2021 Dec 20;17:11200. doi: 10.15766/mep_2374-8265.11200 (PMC8685188; doi:10.15766/mep_2374-8265.11200)
Supplement: Supplementary file 1 — Facilitator Guide.docxAllyship in Residency Module.pptxCase Studies.docxEvaluation Form.docx [file mep_2374-8265.11200-s001.zip › C. Case Studies.docx]

**Allyship in Residency: An Introductory Module on Medical Allyship for Graduate Medical Trainees**

**Case Studies – Participant Version**

**Scenario #1**

**Residency Allyship**

*You are the resident on service working with Dr. Smith. This morning, you have two new medial students working with you on your service that you are just meeting for the first time.*

Scenario:

During morning pre-rounds, you are greeted by two medical students new to your service.. The first medical student introduces themselves and you cordially shake hands. The second medical student states: “Hi, my name is Sam, and my pronouns are she/her/hers”. You greet Sam cordially, and make note of her pronouns.

After a morning of pre-rounding patients, you are joined by your attending, Dr. Smith. Both medical students introduce themselves, and Sam again states her pronouns. However, throughout the course of morning rounds, you notice that the attending keeps referring to Sam as “him” and “his”. Sam politely corrects Dr. Smith the first time, and is met with the response “My apologies, but these days everyone wants to be called something different. It’s hard to keep up”. He continues to misgender Sam for the rest of the day.

**Discuss your role as the Resident in this situation, and how you would choose to approach this scenario.**

**-**Would you choose to address this situation directly on rounds? If so, how?

-If you chose to not address on rounds, how would you address this situation after rounds?

-What are ways to approach this with Sam, the medical student?

-What are ways to approach this with Dr. Smith, the attending?

-How does allyship play a role in this scenario?

**Scenario #2**

**Residency Allyship**

*You are the resident working in a clinic. You are meeting a new patient, Tom, for the first time. Tom is a 10-year-old male. He is accompanied by his mother, Mary.*

Scenario:

This morning in clinic, you notice that a new patient, Tom, is on your schedule. When he arrives, you enter the room and introduce yourself to both Tom and his mother, Mary. Tom anxiously greets you and introduces both himself and his mother. Mary nods silently in your direction.

You ask what has been going on, and why they have come to clinic today, and Tom readily answers all your questions. When you address Mary about what has been going on, she again nods and smiles, and looks over to Tom who interjects and answers again. You begin to suspect that Mary may not be comfortable speaking English.

**Discuss your role as the Resident in this situation, and how you would choose to approach this scenario.**

-How would you approach a discussion with Tom and Mary to assess and discuss Mary’s ability to speak English?

-What would be appropriate next steps for providing care, if Mary is unable to speak English well?

-What would be more appropriate – translator phone or interpreter iPad services? Why?

-What would you do if Tom insists that it is okay for him to remain translating, as that is something he always does?

-How does allyship play a role in this scenario?

-What are additional considerations that may be of concern for this patient?

**Scenario #3**

**Residency Allyship**

*You are the resident working on service, and you have called a consult from your colleague, Dr. Brooks, for your patient, John, with additional concerns. Since you have been with your patient for the past week, you decide to be present for the consultation.*

Scenario

John has been your patient for the last week, and you have noticed that he may have additional health needs that require consultation. John has been a compliant patient and is totally invested in addressing his health needs. You have developed a good rapport with John. You decide to call a consult, and your colleague Dr. Brooks is on service and is able to provide a consult in 15 minutes. You greet Dr. Brooks and enter the room with him.

Upon introduction, John seems visibly tense and his demeanor is notably different than it has been for the last week. John asks if he may have a word alone with you, without Dr. Brooks.

When Dr. Brooks leaves the room, John states “I do not feel comfortable with an African American doctor. I want to be seen by another doctor” You are taken aback, because you have never heard John speak like this before.

**Discuss your role as the Resident in this situation, and how you would choose to approach this scenario.**

-How would you respond to John, after he has told you this information?

-How would you relay this information to Dr. Brooks?

-How does allyship play a role in this scenario?
