# Supplementary material for: Allyship in Residency: An Introductory Module on Medical Allyship for Graduate Medical Trainees
Source: MedEdPORTAL. 2021 Dec 20;17:11200. doi: 10.15766/mep_2374-8265.11200 (PMC8685188; doi:10.15766/mep_2374-8265.11200)
Supplement: Supplementary file 1 — Facilitator Guide.docxAllyship in Residency Module.pptxCase Studies.docxEvaluation Form.docx [file mep_2374-8265.11200-s001.zip › Educational_Summary_Report_11200.pdf]

MedEdPORTAL® The AAMC Journal of Teaching and Learning Resources

Original Publication Open Access

Allyship in Residency: An Introductory Module on Medical Allyship for Graduate Medical Trainees

Sarah Martinez*, Joseph Araj, Symone Reid, MS, Jeslyn Rodriguez, Mytien Nguyen, MS, Dorcas Boahema Pinto, MD, Pamela Y. Young, PA-C, Anicia Ivey, MD, Alexis Webber, MD, Hyacinth Mason, MPH, PhD, CHES

*Corresponding author: martins10@amc.edu

Abstract

Introduction: Lack of diversity impacts research, medical curricula, and medical trainees' ability to provide equitable patient care. The concept of allyship, defined as a supportive association between identities with power and privilege and marginalized identities, provides an optimal framework for enhancing education about health equity. Currently, there are no established curricula focused on allyship and limited mention within current medical training literature. We propose use of allyship to increase graduate medical trainee understanding of diversity and focus on health equity. **Methods:** We developed a 1-hour workshop aimed at helping residents understand the definition of allyship, effective allyship to patients and colleagues, and allyship differences across communities. The workshop consisted of pre- and postassessment surveys, a didactic presentation module, and facilitated case study discussions. It was conducted locally on four occasions across pediatrics, family medicine, surgery, and emergency medicine residency programs. **Results:** An analysis of the 101 preassessment and 58 postassessment survey responses revealed an increased level of knowledge regarding allyship ($p < .001$) and increased comprehension of allyship competencies ($p < .001$). All workshop learning objectives demonstrated positive change postmodule. **Discussion:** With an increasing need for curricula to address health equity in medical trainees, this workshop serves as a unique and effective approach to expanding cultural responsiveness skills under the lens of allyship. Specifically, the workshop functions as a constructive introduction to allyship principles and practices and can serve as a foundation on which residents can build more robust skills as a part of their allyship journey.

Keywords
Allyship, Underrepresented in Medicine, URM, LGBTQ+ Ally, Diversity, Inclusion, Health Equity, Anti-racism

Educational Objectives

By the end of this activity, learners will be able to:

1. Define the term *allyship*.
2. Describe at least one way to be an effective ally towards patients, medical students, and colleagues.
3. Describe how allyship differs across communities.

Introduction

In the last 5 years, medical education has shifted towards an increased focus on diversity, equity, and inclusion. Research shows that lack of diversity significantly influences patient care, medical curricula, and research.[1] In an effort to educate undergraduate medical trainees, a variety of workshops, courses, and training seminars have been created that focus on developing cultural responsiveness skills. While some workshops have been created to fill the gap and further support health equity education, others have worked to decrease implicit biases or increase cultural responsiveness.[2-4] Despite advances for medical students, a lag exists for GME training.

The ACGME sets the standards for residency training curricula and requirements. In its *Common Program Requirements (Residency),* the ACGME has implemented a requirement that residents must meet standards for "advocating for quality patient care and optimal patient care systems."[5] Additionally, it explicitly acknowledes the strength provided by a diverse group of medical trainees.[5] However, even with goals that support diversity, there remains a lack of mandated curricula or courses to best prepare medical graduates to support diverse patient populations and acquire skills specifically intended for that role.

Citation:
Martinez S, Araj J, Reid S, et al. Allyship in residency: an introductory module on medical allyship for graduate medical trainees. *MedEdPORTAL.* 2021;17:11200.
https://doi.org/10.15766/mep_2374-8265.11200

Copyright © 2021 Martinez et al. This is an open-access publication distributed under the terms of the Creative Commons Attribution license.

We believe that one way to address this gap in medical education is by integrating allyship and allyship principles into residency curricula.

Allyship is a term that has gained significant traction in the last decade. It is defined as "supportive association with another group, specifically members of marginalized or mistreated groups to which one does not belong."[6] The Anti-Oppression Network further expands the definition of allyship to include "an active, consistent, and arduous practice of unlearning and re-evaluating, in which a person in a position of privilege and power seeks to operate in solidarity with a marginalized group."[7] Both definitions provide a common language for participants on how to define allyship to further expand the skills of graduate medical trainees as they develop an equitable health care framework.

In medicine, the term *ally* has historically been associated with LGBTQIA+ communities, with many medical schools displaying terms such as *allies, ally,* or *allyship* on their websites in relation to LGBTQIA+ communities.[8-10] Additionally, the limited curricular changes in the literature focused on allyship relate to LGBTQIA+ communities as well.[11] While this association remains important, shifts in health equity as well as experiences and backgrounds of trainees in medical education create a unique opportunity for expansion and use of the term *allyship* for all marginalized communities. Furthermore, in medicine, the intersection of many identities makes this term apt for use expanding beyond standard demographics.

Upon reflection on their clinical experiences, the student developers of this module, each with deep experience serving as leaders in student organizations including the Student National Medical Association, Latino Medical Association, Medical Student Pride Alliance, and Building the Next Generation of Academic Physicians (BNGAP), recognized the important role residents play in facilitating and creating a learning environment that encourages growth in clinical acumen and positively influences career decisions.[12] In response, the student leadership of BNGAP and the Underrepresented Student Alliance at Albany Medical College sought resources. In our review of *MedEdPORTAL,* we found no established curricula focused on allyship. Literature focusing on allyship and how it is applicable to the medical profession remains limited in current graduate medical training curricula.[13-15] Given this, we aimed to fill a void in the GME curriculum by supplying participants with an introductory module on essential allyship competencies for specific communities.

To develop this workshop, we used Kern's six-step curriculum development approach for guidance.[16] First, as part of implementing step 1 of the Kern model, we conducted a general needs assessment, which included our literature review and sharing of medical students' anecdotal experiences. This allowed us to create a targeted needs assessment as outlined in step 2 of Kern's model. The needs assessment was conducted among incoming 2020 family medicine and pediatrics residents at our institution. Results from the Qualtrics-based needs assessment showed a desire for information and formal training on how to best serve patients as well as lack of awareness of the term *allyship* and skills related to allyship towards patients and peers. The general and targeted needs assessments helped guide creation of our learning objectives and goals, which focused on residents' acquisition of knowledge regarding concepts of allyship and medicine, understanding of practical tools for the implementation of allyship principles in medicine, and discussion on forms of allyship to communities that have been historically excluded and are currently underrepresented in medicine. The resulting workshop was created based on findings from our literature review, feedback from the needs assessment, and the anecdotal experiences of the authors. It consisted of a PowerPoint presentation and case discussion. Our goal was to see an increase in the awareness and understanding of allyship principles, practical allyship competencies, and comfort in applying these skills in a clinical setting among participants.

Methods

To meet the practical needs of residency programs at Albany Medical Center in northeast New York, the Allyship in Residency workshop was designed to be conducted in 2 hours, including a 1-hour didactic session and 1-hour case-based discussion. Each session was conducted by a team of medical students from diverse and underrepresented backgrounds in collaboration with our faculty advisor. The senior author, our faculty advisor, was an expert in preventive medicine, health education, and medical education.

The module was offered to residency directors at our institution, and four programs invited us to present to their residents: pediatrics, family medicine, emergency medicine, and surgery. The workshops were scheduled, led, and facilitated by medical students on the team in partnership with residents and residency program directors from the four programs. Residents participated in their respective program's session.

The workshop was implemented a total of four times, once per program, as a part of supplemental didactic learning curricula for residents in the four programs. The workshop consisted of a preassessment survey, a didactic presentation module, facilitated

Copyright © 2021 Martinez et al. This is an open-access publication distributed under the terms of the Creative Commons Attribution license.

case study discussions, and a postassessment survey. This education intervention involved pre- and postworkshop data collection from residents for publication purposes, for which approval was sought and received from the Albany Medical College Institutional Review Board.

Didactic Presentation Module

A medical student from the allyship team and a resident from the respective specialty facilitated a 1-hour didactic presentation. A facilitator guide (Appendix A) was provided to each presenter to guide them through the presentation and case study discussions in a step-by-step manner. The module itself served as a tool with which we were able to ensure that all learners, regardless of previous exposure to allyship principles or lack thereof, were exposed to a robust overview of the topic. The materials needed to give the presentation included the Allyship in Residency module (Appendix B), a computer with Microsoft PowerPoint and HDMI capabilities, and a projector to display the module.

The presentation began by exploring the definitions of diversity, inclusion, and allyship, along with the innate similarities and differences that each of these terms share with one another. We discussed the functional significance of these concepts using examples and statistics relevant to medicine and health care. We then presented participants with content regarding forms of allyship and the interconnecting relationship between allyship and social justice. After we reviewed these critical advocacy and allyship concepts, we transitioned into practical tools for allyship. We employed a functional framework for allyship adapted from Vanderbilt University and focusing on participant introspection and development to assist aspiring resident allies.[17]

Continuing with practical tools for allyship, we discussed specific ways for residents to be allies to members of different underrepresented identities in the medical community, with a focus on skills that could be immediately implemented in daily practice. For the purposes of this workshop, underrepresented in medicine is defined as, but not limited to, LGBTQIA+, Latinx, Indigenous, Black, chronically ill and differently abled, undocumented, female-identifying, non-English speaking, Asian/Asian American, and Muslim communities. The team researched and developed content for the module using resources put forth by respective underrepresented communities. Additionally, student affinity groups at our institution representing each set of communities discussed in the workshop (e.g., Student National Medical Association, Muslim Student Association, etc.) reviewed and provided feedback on content included. While allyship to the aforementioned communities was certainly not limited to the ways discussed in the module, nor was it entirely representative of every member of every community, we tried to provide an accurate snapshot of fundamental skills as they pertained to communities. Guidance for intersectional allyship practices across communities was also provided. The module ended with a discussion on the relevance and importance of this content to the medical team.

Case Studies

Following the module, participants had the opportunity to apply the skills discussed in the presentation to case studies. Learners were divided into groups of five to six and, over the course of an hour, worked through three distinct case studies with a facilitator. The case study coursework (Appendix C) centered around clinical scenarios including misgendering, language barriers, and ethnoracial discrimination. Participants worked together to implement targeted allyship between medical student and resident, patient and resident, and resident colleagues in a safe environment with feedback available.

Evaluation Forms

We administered a pre- and postassessment (Appendix D) to participants prior to and immediately after each didactic lecture. The purpose of this survey was to determine a baseline of preexisting exposure to and understanding of allyship and cultural responsiveness principles discussed in the subsequent presentation, as well as to assess postmodule change in objective and overall knowledge. The presurvey contained demographic information, and the postsurvey included qualitative, feedback-based questions. Participants were allotted approximately 5 minutes to complete the anonymous survey, which was administered using Qualtrics XM software and distributed via a QR code link that could be scanned on mobile devices.

Data Analysis

We analyzed and implemented postworkshop feedback from participants to improve the workshop each successive time it was given. Pre- and postworkshop responses were analyzed using descriptive statistics for demographic factors and participants' responses in allyship situations. The Mann-Whitney *U* test was performed to assess pre- and postworkshop confidence in and knowledge of allyship among survey respondents.

Results

One hundred sixty participants were invited to participate in our workshop. Although we did not confirm attendees, 101 participants completed the presurvey, and 58 completed the postsurvey. While we do not know how many invited attendees participated, based on the assumption that all

Copyright © 2021 Martinez et al. This is an open-access publication distributed under the terms of the Creative Commons Attribution license.

160 invited trainees participated, our response rate was 36%. The participants were spread across four separate sessions within individual residency programs with Albany Medical Center residents from pediatrics, surgery, emergency medicine, and family medicine. Quantitative data were analyzed using the Mann-Whitney *U* test (nonparametric test) to compare pre- and postworkshop results. Qualitative feedback was analyzed through thematic analysis.

One hundred one participants responded to questions assessing participant demographics, and we examined distribution of participant demographics within each identity. We analyzed ethnoracial identity, gender identity, sexual orientation, first-generation status, and Pell Grant reception as a proxy for socioeconomic status. Of the participants who self-reported race and ethnicity in the presurvey, five (5%) identified as Black/African American, one (1%) identified as Latinx/Hispanic, 23 (23%) identified as Asian, six (6%) identified as multiracial, 64 (63%) identified as White, and two (2%) identified as other. Fifty-nine (58%) identified as female, and one (1%) identified as genderqueer. Seventeen participants (17%) were first-generation college graduates, and 18 (18%) were Pell Grant recipients (Table 1). On examination of demographics, our audience consisted of largely majority identities across all metrics, which was representative of residency statistics (Table 1).[18]

Between pre- and postsurvey, we analyzed change in participant responses pre- and postmodule. This analysis included concepts represented in the learning objectives and self-assessed confidence and ability to be an ally. Participants reported a significantly increased level of knowledge regarding the concept of allyship ($p < .001$) and methodologies to be an effective ally towards patients, medical students, and colleagues ($p = .004$; Table 2). Additionally, nearly 10% more participants were able to define allyship after the workshop compared to before the workshop (70% vs. 79%; Table 2). Questions relating to participants' confidence in their ability to be an ally to patients, the importance of allyship training incorporation as part of medical school curricula, and the understanding that allyship looks different amongst varying communities showed no significant differences before and after the workshop (Table 2).

Lastly, we assessed participants' intended response to witnessed discrimination and bias in the work setting. We analyzed pre- and postmodule self-reported intended responses. After the workshop, participants were more likely to report intention to address bias and discrimination in the moment by speaking up or to address the bias/discriminatory behavior later with the person who was biased (Table 3). Fewer participants reported confiding in a colleague or superior/supervisor as their intended method of addressing bias/discrimination after the workshop compared to before the workshop (Table 3).

Table 1. Demographic Information of Participants Who Completed the Preworkshop Survey ($N = 101$)

Demographic Factors	No. (%) of Participants
Race	
Asian/Asian American	23 (23)
Black, African American, African	5 (5)
Latinx, Hispanic	1 (1)
Multiracial	6 (6)
Something else	2 (2)
White, Caucasian	64 (63)
Sex	
Female	59 (58)
Genderqueer or gender nonconforming	1 (1)
Male	41 (41)
Sexual orientation	
Bisexual	3 (3)
Gay	2 (2)
Lesbian	2 (2)
Something else	1 (1)
Straight	91 (92)
Religion	
Agnostic	21 (21)
Atheist	19 (19)
Buddhist	2 (2)
Catholic	20 (20)
Christian	15 (15)
Hindu	8 (8)
Jewish	8 (8)
Muslim	4 (4)
Something else	4 (4)
Differently abled	
Yes	2 (2)
No	98 (98)
First-generation student	
Yes	17 (17)
No	84 (83)
Pell Grant recipient	
Yes	18 (18)
No	83 (82)

Discussion

The Allyship in Residency module was successful in providing an introduction of allyship to graduate medical trainees. Specifically, the biggest impacts were observed regarding introduction of allyship in the medical setting, knowledge regarding the concept of allyship, and participants' intended response to witnessing discrimination and bias in the workplace.

A majority of our participants entered the workshop with a basic preexisting definition of allyship, but it was clear that supplemental knowledge was necessary to translate definition into practice, especially within the medical setting. With further assessment, it became clear that participants expanded their understanding and definition of allyship while also questioning

Copyright © 2021 Martinez et al. This is an open-access publication distributed under the terms of the Creative Commons Attribution license.

Table 2. Participants' Knowledge Regarding Allyship and Confidence in Allyship Abilities

Item	Presurvey	Postsurvey	*p*
Confidence you have in your ability to be an ally to patients, medical students, and colleagues.[a]	$M = 2.7$ ($SD = 0.9$)	$M = 2.9$ ($SD = 0.8$)	.095
Level of knowledge regarding the concept of allyship.[b]	$M = 2.5$ ($SD = 0.9$)	$M = 3.2$ ($SD = 0.7$)	<.001[c]
Allyship training and cultural competency training should be a required part of medical school curriculum.[d]	$M = 4.3$ ($SD = 1.0$)	$M = 4.5$ ($SD = 0.9$)	.266
Definition of allyship.	70%[e]	79%[e]	.199
Ways to be an effective ally towards patients, medical students, and/or colleagues.	67%[e]	88%[e]	.004[c]
What does allyship look like across different communities?	92%[e]	98%[e]	.154

[a]Rated on a 5-point Likert scale (0 = *no confidence*, 4 = *complete confidence*).
[b]Rated on a 5-point Likert scale (1 = *not knowledgeable at all*, 5 = *extremely knowledgeable*).
[c]Significant at $p < .05$.
[d]Rated on a 5-point Likert scale (1 = *strongly disagree*, 5 = *strongly agree*).
[e]Percentage of participants selecting the correct answer.

the limited nature of a simplified definition. Allyship is a term that has not traditionally been used in a medical context, so when used as a metric for health equity, it may require additional considerations.[19] It is unclear whether the majority of participants entered the workshop with a basic idea of what allyship looked like across different communities as question phrasing may have influenced participants' ability to answer knowledge questions correctly. Regardless, the ultimate goal was to improve knowledge on allyship, and we feel this was accomplished and created significant impact. One participant provided the following feedback: "It was a good introduction to Allyship. It was important to us because many of us have no exposure to Allyship." Additional feedback expressed a common theme that the sessions provided important and thought-provoking information that encouraged participants to expand their understanding. Given the lack of preexisting literature on allyship in medical education, this information may serve to better indicate where the general baseline of knowledge exists and where supplemental initiatives and education should be targeted. At the very least, feedback reflected that participants placed allyship as a consideration in medical settings, a necessity for effecting further change.

When participants were asked how they would respond to witnessed discrimination or bias in the medical setting, their answers reflected an intention to respond more actively. This shift supports a correlation of knowledge and comfort level with active allyship and intervention. Feedback reflected this, as one participant reported, "I like that it encouraged us to go deeper than 'knowing something.' That is a step, but I feel more empowered to take it further." Higher levels of intentions combined with the knowledge and understanding of the concept of allyship can be effective in transforming knowledge into action, the ultimate goal of this workshop. It has been shown that true allyship requires active participation of allies along with elimination of the bystander effect.[20] We feel that our workshop, through introductory education, can serve as a stepping-stone to more targeted and active practices.

Table 3. Preworkshop Versus Postworkshop: How Participants Intend to Respond When They Witness Bias or Discrimination

	No. (%) of Participants	
**Response**	**Presurvey**	**Postsurvey**
Address later to person who experienced bias	6 (6)	2 (4)
Address later to person who displayed biased behavior	10 (11)	12 (21)
Address in the moment by speaking up	25 (27)	21 (37)
Confide in a colleague	28 (30)	10 (18)
Confide in a superior/supervisor	15 (16)	7 (12)
Ignore it	7 (8)	3 (5)
Other	2 (2)	2 (4)
Total	93 (100)	57 (100)

Despite improvement in knowledge and responses, participants' confidence in their ability to be an ally to patients, medical students, and colleagues showed minimal improvement, which is disheartening but provides better understanding of how to leverage the module for optimal education. This workshop is intended to serve as an introduction to allyship, and while it offers basic principles and skills, it is not meant to provide more advanced, clinically specific skills or knowledge. Additionally, focused, in-depth allyship content on each population represented in the presentation is not provided. While we considered decreasing the number of communities represented, we felt it was necessary to introduce a breadth of identities so that participants could appreciate that allyship is not limited to only a few identities. In fact, feedback reflected the desire for inclusion of more communities than we presented. Our hope is that participants will be able use the resources provided to build on the foundation established in the workshop and continue to expand their allyship competencies for specific communities beyond the scope of what we are able to discuss.

Limitations

The study was conducted with programs that tend to skew more female and reflects the composition of the residencies. Although

Copyright © 2021 Martinez et al. This is an open-access publication distributed under the terms of the Creative Commons Attribution license.

our demographics were 63% White and 58% female, we believe that the results are generalizable given the breadth of the topics covered, which range from ethnicity and gender to ability. Our response rate of 36% was disappointing, but we found from a search of *MedEdPORTAL* that other responses have ranged from 13% to 100% and we fell within that range.[21-23]

In all four residency programs that implemented this workshop, residents at all levels of training were invited to attend. Ideally, residents should receive this training as part of orientation or as early as possible in their residency. This type of workshop could serve as the foundation for a longitudinal program to build and practice allyship skills throughout the entire course of graduate education. Resident days are busy, and finding opportunities to present the workshop can be challenging.

Given the expansive nature of this topic and the identities discussed, we found ourselves constrained by the level and amount of education we could provide within a limited time frame. The module was designed to be a general introductory session presenting a discussion of allyship in a GME environment. We felt that given the intersectionality of many of these identities, it was important to expand beyond one to two. Many of the identities we chose to represent often have not been included in discussions surrounding allyship. It was important to us to help residents adopt an allyship lens and understand the concept as it relates to the intersectionality of the patients they serve and the colleagues they work with. We intentionally developed the curriculum to be flexible enough to be adapted and tailored to meet the needs of a variety of learning settings and contexts.

We believe that there is a need for long-term, longitudinal curricula focused on allyship to facilitate continued growth, knowledge acquisition, and clinical skill training. Our worksop is designed to serve as an introduction in service of one of the core tenets of allyship it discusses—allyship as a continuous process that requires sustained, arduous learning and practice, as opposed to a state of being or an end goal that can be achieved following a single workshop. However, for those with limited prior exposure to allyship in their medical education, as we have found is often the case, the workshop is an ideal place to introduce this process. We believe that in addition to implementing this single-session workshop, it is important for residency programs to create opportunities for residents to fully develop and expand their allyship skills in practice.

Additionally, prior to the training, facilitators may not have been familiar with the tools presented or all the communities represented. For example, prior knowledge about specific communities may have altered the experience presented to residents between residency programs. Although our facilitator guide is flexible enough to train anyone to provide the presentation, we found the best facilitators were those who themselves were appraised by others as allies and were invested in working with marginalized populations. Supplemental facilitator training may help to strengthen the experience and knowledge base of facilitators, thereby enhancing the workshop for trainees as a natural consequence.

We recognize that the case scenarios are not representative of all the communities discussed in the workshop. Specific community-focused training as part of a larger longitudinal curriculum would help alleviate this gap in knowledge acquisition.

Our findings also highlight the need for future workshops aimed at providing skills and further focused education regarding different communities and clinical allyship skill building. Integrating this workshop with the standardized patient program is one way to further increase resident comfort surrounding these topics. Future iterations could include opportunities for residents to practice allyship in a standardized format.

Lastly, when implementing this workshop, future facilitators and presenters should understand that it is intended for introductory knowledge building. Each identity represented is far more complex than shown, and all are deserving of workshops solely dedicated to educating about the complexities of their culture and allyship-related considerations. For next steps, a deeper dive into a more longitudinal curriculum targeted towards exploration of each individual group should occur. Ideally, this will increase nuances and depths of allyship within each community represented in the introductory module, thereby continuing to facilitate conversations and allowing participants to evolve in their allyship journey.

Appendices

A. Facilitator Guide.docx

B. Allyship in Residency Module.pptx

C. Case Studies.docx

D. Evaluation Form.docx

All appendices are peer reviewed as integral parts of the Original Publication.

Copyright © 2021 Martinez et al. This is an open-access publication distributed under the terms of the Creative Commons Attribution license.

Sarah Martinez: Third-Year Medical Student, Albany Medical College; ORCID: https://orcid.org/0000-0002-8050-0798

Joseph Araj: Second-Year Medical Student, Albany Medical College

Symone Reid, MS: First-Year Medical Student, Albany Medical College

Jeslyn Rodriguez: Second-Year Medical Student, Albany Medical College

Mytien Nguyen, MS: MD/PhD Candidate, Yale School of Medicine

Dorcas Boahema Pinto, MD: Assistant Professor and Clerkship Director, Department of Emergency Medicine, Albany Medical Center

Pamela Y. Young, PA-C: Director of Advanced Practice Provider Operations, Department of Emergency Medicine, Albany Medical Center

Anicia Ivey, MD: Resident, Department of Family Medicine, Albany Medical Center

Alexis Webber, MD: Resident, Department of General Surgery, Albany Medical Center

Hyacinth Mason, MPH, PhD, CHES: Assistant Dean of Student Affairs, Tufts University School of Medicine

Acknowledgments
We thank the Underrepresented Student Alliance, the Student National Medical Association chapter at Albany Medical College (AMC), the Building the Next Generation of Academic Physicians AMC chapter, and the Latino Medical Association chapter at AMC; Dr. Branden Eggan for her support throughout this process and comments on early drafts; Dr. Angela Antonikowski for her review of questions during the resident needs assessment phase of this work; Tanuj Sharma, BS, for his advice and support throughout this process; and Angela T. Sweeney, MEd, MA, for her guidance and review of early drafts.

Disclosures
None to report.

Funding/Support
None to report.

Ethical Approval
The Albany Medical College Institutional Review Board approved this project.

References

1. Kirch DG. Addressing racism and mistreatment in academic medicine. Association of American Medical Colleges. February 21, 2019. Accessed September 9, 2021. https://www.aamc.org/news-insights/addressing-racism-and-mistreatment-academic-medicine
2. Brooks KC, Rougas S, George P. When race matters on the wards: talking about racial health disparities and racism in the clinical setting. *MedEdPORTAL*. 2016;12:10523. https://doi.org/10.15766/mep_2374-8265.10523
3. DallaPiazza M, Padilla-Register M, Dwarakanath M, Obamedo E, Hill J, Soto-Greene ML. Exploring racism and health: an intensive interactive session for medical students. *MedEdPORTAL*. 2018; 14:10783. https://doi.org/10.15766/mep_2374-8265.10783
4. Holm AL, Rowe Gorosh M, Brady M, White-Perkins D. Recognizing privilege and bias: an interactive exercise to expand health care providers' personal awareness. *Acad Med*. 2017; 92(3):360-364. https://doi.org/10.1097/ACM.0000000000001290
5. Accreditation Council for Graduate Medical Education. *ACGME Common Program Requirements (Residency)*. Accreditation Council for Graduate Medical Education; 2021. Accessed September 9, 2021. https://www.acgme.org/Portals/0/PFAssets/ProgramRequirements/CPRResidency2021.pdf
6. Allyship. Merriam-Webster. Accessed September 9, 2021. https://www.merriam-webster.com/dictionary/allyship
7. Allyship. Anti-Oppression Network. Accessed September 9, 2021. https://theantioppressionnetwork.com/allyship/
8. LGBTQ and Allies at Harvard Medical School. Harvard University. Accessed September 9, 2021. https://gsas.harvard.edu/student-life/harvard-resources/lgbtq-and-allies-harvard-medical-school
9. LGBTQ students and allies. University of Michigan Medical School. Accessed September 9, 2021. https://medicine.umich.edu/medschool/education/md-program/our-community/students-faculty/lgbtq-students-allies
10. *Ally Campaign: Self-Guided Training for Allies on the Health Sciences Center*. LGBT Center at the University of Louisville; 2015. https://louisville.edu/lgbt/hsc/ally-campaign-at-the-health-sciences-center-campus/AllyCampaignSelfguidedtrainingFINAL.pdf
11. Grova MM, Donohue SJ, Bahnson M, Meyers MO, Bahnson EM. Allyship in surgical residents: evidence for LGBTQ competency training in surgical education. *J Surg Res*. 2021;260:169-176. https://doi.org/10.1016/j.jss.2020.11.072
12. Musunuru S, Lewis B, Rikkers LF, Chen H. Effective surgical residents strongly influence medical students to pursue surgical careers. *J Am Coll Surg*. 2007;204(1):164-167. https://doi.org/10.1016/j.jamcollsurg.2006.08.029
13. Mensah MO. Majority taxes—toward antiracist allyship in medicine. *N Engl J Med*. 2020;383(4):e23. https://doi.org/10.1056/NEJMpv2022964
14. Bullock JL, Lockspeiser T, del Pino-Jones A, Richards R, Teherani A, Hauer KE. They don't see a lot of people my color: a mixed methods study of racial/ethnic stereotype threat among medical students on core clerkships. *Acad Med*. 2020;95(11)(suppl): S58-S66. https://doi.org/10.1097/ACM.0000000000003628
15. Nixon SA. The coin model of privilege and critical allyship: implications for health. *BMC Public Health*. 2019;19:1637. https://doi.org/10.1186/s12889-019-7884-9
16. Robertson AC, Fowler LC, Niconchuk J, et al. Application of Kern's 6-step approach in the development of a novel

Copyright © 2021 Martinez et al. This is an open-access publication distributed under the terms of the Creative Commons Attribution license.

anesthesiology curriculum for perioperative code status and goals of care discussions. *J Educ Perioper Med*. 2019;21(1):E634.

17. Lucci S, Hua J. Allyship. Presented at: Talk About It Thursdays; April 7, 2016; Vanderbilt University, Nashville, TN.
18. Accreditation Council for Graduate Medical Education. *Data Resource Book: Academic Year 2018-2019*. Accreditation Council for Graduate Medical Education; 2019.
19. Allyship: what it means to be an ally. Tulane University School of Social Work. November 18, 2019. Accessed September 9, 2021. https://socialwork.tulane.edu/blog/allyship
20. Salter NP, Migliaccio L. Allyship as a diversity and inclusion tool in the workplace. In: Georgiadou A, Gonzalez-Perez MA, Olivas-Luján MR, eds. *Diversity Within Diversity Management: Types of Diversity in Organizations*. Emerald Publishing; 2019:131-152.
21. Borowsky H, Morinis L, Garg M. Disability and ableism in medicine: a curriculum for medical students. *MedEdPORTAL*. 2021;17:11073. https://doi.org/10.15766/mep_2374-8265.11073
22. Bowler C, Foshee C, Haggar F, Simpson D, Schroedl C, Billings H. Got 15? Try Faculty Development on the Fly: a snippets workshop for microlearning. *MedEdPORTAL*. 2021;17:11161. https://doi.org/10.15766/mep_2374-8265.11161
23. Wulf KL, Hurtubise L, Brod H, Binkley PF. The CARE Inventory: a self-reflective, behavior-based instrument to guide professional development and mentorship of academic faculty. *MedEdPORTAL*. 2018;14:10763. https://doi.org/10.15766/mep_2374-8265.10763

Received: March 28, 2021
Accepted: August 31, 2021
Published: December 20, 2021

Copyright © 2021 Martinez et al. This is an open-access publication distributed under the terms of the Creative Commons Attribution license.
